# Sub-Inhibitory Fosmidomycin Exposures Elicits Oxidative Stress in *Salmonella enterica* Serovar *typhimurium LT2*

**DOI:** 10.1371/journal.pone.0095271

**Published:** 2014-04-21

**Authors:** David T. Fox, Emily N. Schmidt, Hongzhao Tian, Suraj Dhungana, Michael C. Valentine, Nicole V. Warrington, Paul D. Phillips, Kellan B. Finney, Emily K. Cope, Jeff G. Leid, Charles A. Testa, Andrew T. Koppisch

**Affiliations:** 1 Bioscience Division, Los Alamos National Laboratory, Los Alamos, New Mexico, United States of America; 2 Department of Chemistry, Northern Arizona University, Flagstaff, Arizona, United States of America; 3 Department of Biology, Northern Arizona University, Flagstaff, Arizona, United States of America; 4 Echelon Biosciences, Inc, Salt Lake City, Utah, United States of America; University of Louisville, United States of America

## Abstract

Fosmidomycin is a time-dependent nanomolar inhibitor of methylerythritol phosphate (MEP) synthase, which is the enzyme that catalyzes the first committed step in the MEP pathway to isoprenoids. Importantly, fosmidomycin is one of only a few MEP pathway-specific inhibitors that exhibits antimicrobial activity. Most inhibitors identified to date only exhibit activity against isolated pathway enzymes. The MEP pathway is the sole route to isoprenoids in many bacteria, yet has no human homologs. The development of inhibitors of this pathway holds promise as novel antimicrobial agents. Similarly, analyses of the bacterial response toward MEP pathway inhibitors provides valuable information toward the understanding of how emergent resistance may ultimately develop to this class of antibiotics. We have examined the transcriptional response of *Salmonella enterica* serovar *typhimurium* LT2 to sub-inhibitory concentrations of fosmidomycin via cDNA microarray and RT-PCR. Within the regulated genes identified by microarray were a number of genes encoding enzymes associated with the mediation of reactive oxygen species (ROS). Regulation of a panel of genes implicated in the response of cells to oxidative stress (including genes for catalases, superoxide dismutases, and alkylhydrogen peroxide reductases) was investigated and mild upregulation in some members was observed as a function of fosmidomycin exposure over time. The extent of regulation of these genes was similar to that observed for comparable exposures to kanamycin, but differed significantly from tetracycline. Furthermore, *S. typhimurium* exposed to sub-inhibitory concentrations of fosmidomycin displayed an increased sensitivity to exogenous H_2_O_2_ relative to either untreated controls or kanamycin-treated cells. Our results suggest that endogenous oxidative stress is one consequence of exposures to fosmidomycin, likely through the temporal depletion of intracellular isoprenoids themselves, rather than other mechanisms that have been proposed to facilitate ROS accumulation in bacteria (e.g. cell death processes or the ability of the antibiotic to redox cycle).

## Introduction

**Figure 1:**
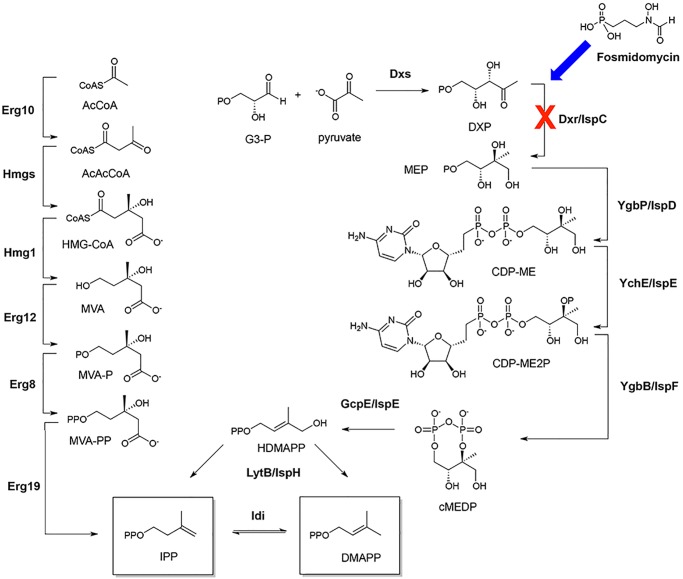
Biosynthesis of isopentenyl diphosphate (IPP) and dimethylallyl diphosphate (DMAPP). IPP and DMAPP are formed through the mevalonate pathway (left) in mammals, fungi and plants and through the methylerythritol phosphate (MEP) pathway (right) in many bacteria, green algae, and plant chloroplasts. Fosmidomycin inhibits formation of IPP and DMAPP (and thus late stage isoprenoid compounds) through disruption of the MEP pathway.

Fosmidomycin is an inhibitor of methylerythritol phosphate (MEP) synthase, which is the enzyme responsible for the first committed step in the biosynthesis of the isoprenoid precursors isopentenyl diphosphate (IPP) and dimethylallyl diphosphate (DMAPP) via the MEP pathway (Figure 1) [1]–[4].

The MEP pathway is the sole route to isoprenoids in most bacteria, including mycobacteria, Gram-negative and Gram-positive strains, in addition to some eukaryotic parasites. Given that the enzymes responsible for the biosynthesis of isoprenoids are required for bacterial proliferation, and there are no human enzyme orthologs, the MEP pathway has emerged as an attractive target for the development of new broad spectrum antimicrobial agents.[5]–[7] While fosmidomycin and its interaction with MEP synthase has been thoroughly investigated,[1]–[3] much less is understood about the coordinated metabolic pathways bacterial cells evoke upon exposure to the antibiotic. Further, a report by Odom and coworkers documented that at higher concentrations fosmidomycin may also target a second MEP pathway-specific enzyme.[8] This study serves as a prime example that, despite several decades of research into the compound, much information remains to be discovered.

Genome-wide transcriptional profiling via DNA microarray analysis was employed to investigate a bacterium’s response to sub-inhibitory concentrations of antibacterial agents. Transcriptional analysis is useful both as an aid to the process of determining mechanisms of action of novel compounds [9], [10] as well as garnering a greater understanding of the intrinsic metabolic response of bacteria toward dilute concentrations of antibiotics [11]–[14]. Similarly, differential proteomics has proven useful in analyses of the metabolic response of bacteria toward antibiotics. As part of our long-standing interest in MEP pathway inhibitors as anti-infective agents [2], [7], [15], we have pursued differential proteomic analyses on multiple bacteria, including *Salmonella enterica* serovar *typhimurium*, exposed to sub-inhibitory concentrations of fosmidomycin and related MEP pathway-specific inhibitors. Further, we have performed the identical studies on a MEP synthase knockout *S. typhimurium* strain (**Δ** *dxr*). In the absence of exogenously supplied methylerythritol (ME) this strain is unable to biosynthesize isoprenoids and thus was expected to have a similar response to the wild-type strain when exposed to MEP pathway-specific inhibitors. Our initial studies indicated that among the proteins regulated by MEP pathway inhibition were several enzymes associated with ROS detoxification and oxidative stress. In order to compile a more comprehensive analysis of the metabolic response of *S. typhimurium* to fosmidomycin, we also chose to examine the bacterium’s transcriptional response via microarray.

Although the intrinsic response of bacteria to antibiotics is complex and multi-faceted, oxidative stress as mediated by the exposure to antibiotics (more specifically, the formation of intracellular ROS upon exposure of cells to antibiotics) has been the focus of a significant amount of recent research. Interestingly, in recent reports, there are postulations that diverse bactericidal antibiotics (β-lactams, aminoglycosides and fluoroquinolones), despite having discrete intracellular targets, facilitate cell death via a common mechanism involving the indirect production of ROS with subsequent lethal damage to DNA and other cellular components [16]–[23]. In this mechanism, upon antibiotic exposure, the tricarboxylic acid (TCA) cycle is upregulated followed by activation of the electron transport chain.[24] ROS generated in this process is postulated to destabilize iron-sulfur cluster containing proteins, and ultimately result in an increase in intracellular Fe (II) concentrations that, along with hydrogen peroxide, serves as a substrate for the generation of hydroxyl radicals via the Fenton reaction.[19] Similarly, ROS generated from sub-lethal exposures of bactericides were proposed to facilitate the emergence of antibiotic resistance via radical-induced mutagenesis.[25] This hypothesis, however, has been the subject of debate. In two independent reports, Lui and Imlay and Lewis and coworkers contend that bactericidal antibiotics either do not elicit ROS production, or they elicit production to levels that are insufficient to affect cell killing.[26], [27] In these reports, little correlation was observed between antibiotic efficacy and oxygen availability in (or oxygen consumption from) the environment. Moreover, intracellular hydrogen peroxide generation in cells proffered canonical antibiotics was observed to be far lower than those exposed to paraquat, which is a redox-cycling antibiotic known to generate ROS.[27] Very recently, two reports were communicated that demonstrate that ROS can be influential in antibiotic treatments.[28], [29] Accumulation of intracellular ROS in *Escherichia coli* was reported to potentiate the action of various bactericides against this strain, and silver was shown to sensitize Gram-negative strains to vancomycin through elicitation of oxidative stress networks.

The goal of this work was twofold. First, we have examined the transcriptional profile of *S. enterica* serovar *typhimurium* LT2 exposed to sub-inhibitory concentrations of fosmidomycin via cDNA microarray to analyze genes regulated by a short exposure to dilute concentrations of this antibiotic. Second, we have further investigated transcription of genes encoding common ROS mediating enzymes as a function of exposure time to sub-inhibitory doses of fosmidomycin relative to untreated controls as well as comparable concentrations of other more conventional antibiotics as a means to assess the relative intracellular oxidative stress incurred upon exposure to these compounds.

## Results

### Changes in the Global Gene Expression Induced by Fosmidomycin

As mentioned earlier, our initial investigation into the metabolic response of MEP pathway disruption in *S. typhimurium* was conducted through a differential proteomics analysis of a conditional mutant. In these studies, we exploited the *S. typhimurium* strain CT12, which lacks a functional *dxr* gene due to chromosomal disruption and thus is dependent upon exogenous supplementation of methylerythritol (ME). CT12 imports and phosphorylates ME with an ATP-dependant kinase to provide MEP, thereby reconstituting a functional pathway for isoprenoid synthesis and growth.[30] After 45 minutes of continued growth following removal of ME from the culture medium of mid-logarithmic propagating *S. typhimurium* CT12, numerous enzymes generally associated with redox processes or oxidative stress were upregulated. Some examples include alkylhydrogen peroxide reductase, superoxide dismutase, and several peroxidases, among others (Table S2). Several of these same enzymes were also observed to be upregulated after short (30 min) exposures of mid-logarithmic propagating wild-type *S. typhimurium* LT2 cells to 10 **µ**g/mL fosmidomycin (which represents 0.5X of the minimal inhibitory concentration calculated in our laboratory under comparable experimental conditions) (Table S3). To investigate this phenomenon more closely, similar antibiotic challenges were conducted for a shorter interval (20 min) for analysis via microarray. In these tests, 493 total genes were observed to be regulated with statistical significance (173 genes with a p<0.01 as measured with a Student’s t-test and a further 320 genes with a p<0.05) which represent approximately 11% of the total genes. Overall, the regulation observed under these conditions is relatively mild across the genome, and as is common in this type of experiment, the transcription of genes associated with many types of metabolic processes are influenced by the introduction of antibiotic.[11], [12], [31]–[34]. Of the identified genes, the levels of 277 (6.2% of the total genes) were perturbed by 50% or more relative to an untreated control (≥1.5 or ≤0.67 fold change upon introduction of fosmidomycin; Table S4), which reflects relatively minor regulation overall. Any global profile of regulated genes reflects transcripts isolated from a population of cells experiencing the same conditions (in this case, antibiotic exposure), however it is possible that sub-populations within this group may exist that respond to the conditions dramatically differently than the average. Specifically, a heterogeneous response to fosmidomycin across the population, wherein a small sub-population of cells are strongly affected (and perhaps inhibited/killed by the antibiotic) in the midst of many others that are largely unaffected would also give rise to mild average regulation overall. While we cannot definitively conclude that a robustly responsive subpopulation is not influencing the regulation we observe, we did not observe statistically significant differences in the ratio of live to dead cells in fosmidomycin exposures compared to untreated controls over the course of our assay using commercially available fluorescent stains (Figure S1). The upregulated genes were significantly represented by genes classified as those involved in carbohydrate utilization, amino acid transport and metabolism, fatty acid catabolism, protein folding, DNA transcription and regulation, general transport, and electron transport/oxidative stress (Table 1). For the most part, the regulated genes we observe in our transcriptional experiments and the proteins observed in our differential proteomics experiments belong to common pathways, however there is much less correlation of specific genes/proteins between the two sets. There are a number of factors that could contribute to this observation. First, one of our proteomic experiments was conducted by depleting the isoprenoids of a ME auxotrophic strain as opposed to antibiotic-mediated isoprenoid depletion with fosmidomycin. Starvation of the auxotroph should ultimately elicit a phenomenon comparable, but not identical to, inhibition of the MEP pathway with fosmidomycin, and as such we did not expect the transcriptional and proteomic profiles to overlap perfectly. However, differences in overlap of the two profiles are not uncommon even within more closely related experimental sets (e.g. fosmidomycin exposure). It is common for differential proteomics experiments to report quantifiable differences for expressed proteins that correlate to a subset of all regulated genes due to experimental limitations of the protein identification process. In differential proteomics experiments like those employed here, crude protein fractions are isolated from cells and each individual component protein must be first purified from this mixture prior to identification via MS. Some proteins simply exist in solution at a low enough abundance that identification alone is challenging. Another contributing factor may be that the time points selected for harvesting of transcripts and proteins, respectively, do not overlap perfectly. For instance, many of the genes observed to be regulated at 20 minutes may not be transcribed to proteins in comparable concentrations after 30 minutes. Conversely, some genes that may no longer be upregulated at 20 minutes post exposure may result in accumulation of a significant amount of protein after 30 minutes. Given these experimental constraints, we believe the regulation of common pathways in our transcriptional and proteomic analyses is a significant observation. Upregulation of many transporters, chaperones and transcriptional elements is largely regarded as a general response to bacterial interrogation by antibiotics as an attempt to avoid their deleterious effects. Under the conditions of this assay, little regulation of genes in the MEP pathway itself was observed (only *ipk*, also known as *ispE*, was observed to be mildly downregulated), nor were regulation of any genes previously implicated in the development of fosmidomycin resistance (*glpT*, *fsr*)[35]–[38]. A significant component of the genes upregulated by fosmidomycin exposure are catabolic in nature and are presumably involved in carbon utilization either from carbohydrates, fatty acids or amino acids. Under these conditions, little regulation is observed in glycolytic or TCA cycle enzymes. However, almost all of the upregulated genes within amino acid metabolism act upon glucogenic amino acids such as glutamine, arginine or histidine, which may indicate redirection of carbon flow from the respective amino acids to these energy generating pathways. Interestingly, indole, which is a product of the initial reactions in tryptophan catabolism, is observed to accumulate in high levels in *E. coli* strains engineered to overproduce isoprenoids.[39] We do not observe tryptophan catabolism under these conditions in *S. typhimurium*, however, we have observed tryptophanases to be upregulated in similar tests of *E. coli* challenged with fosmidomycin (data not shown). Taken together, these observations are suggestive that amino acid catabolism, at least in *S. typhimurium* and related bacteria, is initiated in response to a strain on the MEP pathway.

**Table 1:** Categories of regulated genes observed upon sub-inhibitory exposure to fosmidomycin.

	Observed Regulation	
Gene category	Up	Down
**Transporters**	21	22
Sugar transporters	4	6
Amino acid transporters	13	3
Inorganic ion/heme transporters	0	8
Other transporters	4	5
**Carbohydrate metabolism**	11	5
**Amino acid metabolism**	8	4
**Fatty acid metabolism**	6	0
**DNA replication, recombination and repair**	7	12
Transcriptional regulators	6	7
DNA/RNA modification	0	2
DNA repair/recombination	1	4
**Cell wall/membrane biogenesis**	4	19
**Aerobic respiration/oxidative stress**	11	2
Electron transport chain	8	2
ROS mediation	3	0
**Anaerobic respiration**	1	23
Nitrate utilization	0	12
Organic acid utilization	1	3
Thiol utilization	0	4
Hydrogenases	0	4
**Protein folding, modification, and repair**	8	2
Chaperones	4	2
Proteases	2	0
Enzyme repair	2	0
**Cofactor biosynthesis**	3	10
**Chemotaxis**	0	7
**Cell motility**	0	28
**Virulence/invasion**	0	9
**Secreted proteins**	0	5
**Function unknown**	9	19
**Other**	3	18

All assignments were made based on analysis of identified genes using the Kyoto Encyclopedia of Genes and Genomes (KEGG) database (www.genome.jp/kegg/).

Conversely, the downregulated genes were largely represented by those reflecting cell motility, nitrite, thiol and organic acid metabolism, transporters, chemotaxis, cofactor biosynthesis, and cell wall composition/modification. Furthermore, numerous genes with a putative role in virulence, invasion or predicted to encode secreted proteins were downregulated. An increase in flagellation and expression of virulence genes is a characteristic of a swarm cell phenotype in many bacteria, including *S. typhimurium*.[40]–[44] Actively swarming cells were observed to show an elevated resistance to various antibiotics and ROS through overproduction of cysteine-based antioxidants (relative to their swim cell counterparts).[45], [46] Coordinated repression of flagellar biosynthesis genes and virulence-associated genes is suggestive that fosmidomycin may be influencing metabolic networks normally associated with swim/swarm differentiation in some way; however, repression of these aforementioned genes is counter to what is observed upon development of the swarm state. Furthermore, under these conditions we do not observe regulation of cysteine biosynthesis genes, which has also been shown to be critical for the swarm phenotype in *Salmonella*.[46] Thus, we believe the upregulation observed in ROS mediating genes (discussed below) is likely independent of swim/swarm processes even though upregulation of these types of genes is common in the swarm phenotype relative to the swim phenotype. Other downregulated genes were largely comprised of genes encoding cell wall associated proteins or enzymes associated with anaerobic utilization of nitrate, small organic acids (formate) or thiols (dimethylsulfoxide). The downregulated transporters were largely comprised by phosphotransferase-type (PTS) permeases and those responsible for inorganic ions and metals.

Due to their implication in interactions with bacteria with other bactericidal antibiotics, particular interest was noted for the upregulated genes encoding enzymes involved in the electron transport chain or those responsible for ROS mediation. In addition to genes encoding respiratory enzymes (such as *cyoA*, *cyoB* and *sdhD*), fosmidomycin induced mild expression of genes with roles in the response to and mediation of ROS (Table 2), among them *soxS*, *sodA*, and *ahpF*. *sodA* encodes superoxide dismutase, which reduces superoxide to hydrogen peroxide and oxygen, *ahpF* encodes one of two components of alkylhydrogen peroxide reductase, which is a primary defense enzyme used by bacteria to detoxify hydrogen peroxide [47], and *soxS* is one of the primary transcriptional regulators in *Salmonella* activated to respond to oxidative stress. *soxS* coordinates expression of several genes (including *sodA*) that encode enzymes that ameliorate ROS or repair Fe-S clusters. The regulation observed via microarray was consistent with the hypothesis that fosmidomycin exposure may be inducing some degree of oxidative stress to the cells.

**Table 2:** Regulation of selected genes of relevance to oxidative stress identified in microarray analysis of short (20 min) sub-inhibitory exposures to fosmidomycin or kanamycin.

Gene number	Gene name	Enzyme encoded	Fosmidomycin exposureFold regulation (p value)	Kanamycin exposureFold regulation (p value)
STM0443	*cyoA*	Cytochrome o ubiquinoloxidase subunit II	4.10 (0.003)	4.59 (0.001)
STM0442	*cyoB*	Cytochrome o ubiquinoloxidase subunit I	3.93 (0.003)	3.58 (0.001)
STM4265	*soxS*	DNA-binding transcriptionalregulator soxS	3.51 (0.001)	0.99 (0.955)
STM4055	*sodA*	Superoxide dismutase	3.02 (0.007)	6.78 (0.004)
STM0733	*sdhD*	Succinate dehydrogenasecytochrome b556 small subunit	1.85 (0.021)	1.8 (0.006)
STM0441	*cyoC*	Cytochrome o ubiquinoloxidase subunit III	1.78 (0.004)	1.44 (0.061)
STM0732	*sdhC*	Succinate dehydrogenasecytochrome b556 large subunit	1.67 (0.008)	1.51 (0.010)
STM0609	*ahpF*	Alkyl hydroperoxidereductase F52a subunit	1.57 (0.006)	1.56 (0.001)

Interestingly, some similarities in the profile of genes regulated upon fosmidomycin exposures were also observed upon comparable microarray analysis of kanamycin exposures to *S. typhimurium* (Table 2). As noted previously, the phenomenon of bactericide mediated oxidative stress is the source of substantial debate in the literature, and the phenomenon itself has not been hypothesized to occur upon exposure to bacteriostatic agents (such as fosmidomycin at lower concentrations [32]). Nonetheless, the overlap in the transcriptional profile of these two examined antibiotics led us to investigate whether cells exposed to fosmidomycin may be experiencing a metabolic phenomenon akin to that elicited by kanamycin. For a further comparison, similar analyses were also performed on cells exposed to ampicillin (bactericide) and tetracycline (bacteriostat).

### Sub-inhibitory Exposures to Antibiotics and qRT-PCR Timecourse of Genes Involved in ROS Mediation

**Figure 2:**
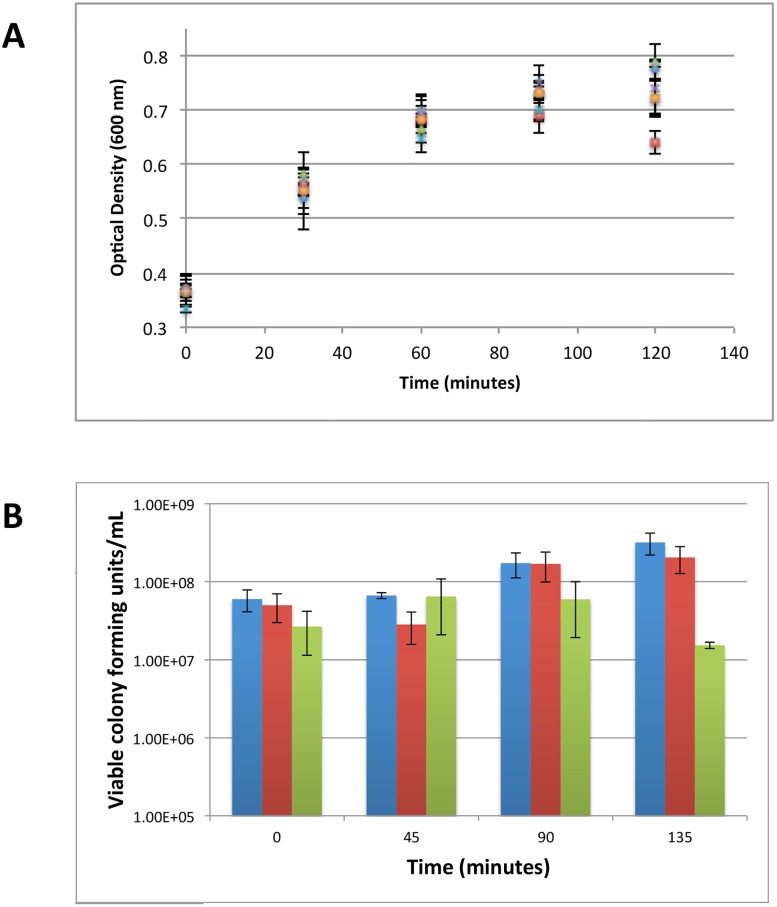
Effect of antibiotic exposure on cells in mid-logarithmic growth. A) Cell viability measured by OD_600_ vs time (Key:+: no antibiotic, blue diamond: 1 **µ**g/mL ampicillin, red square: 10 **µ**g/mL ampicillin, green triangle: 2 **µ**g/mL kanamycin, purple cross: 20 **µ**g/mL kanamycin, blue dash: 20 **µ**g/mL fosmidomycin, orange circle: 200 **µ**g/mL fosmidomycin) B) Cell viability measured in viable colony forming units/mL for a representative bactericide (ampicillin) exposure (Key: blue:no antibiotic, red: 1 **µ**g/mL ampicillin, green: 10 **µ**g/mL ampicillin). All values represent the average of at least three independent biological replicates and the error reflects the standard error of the mean.

Prior to investigation of the exposures with RT-PCR, it was necessary to verify that the concentrations of antibiotics in our assay were truly representative of sub-inhibitory/sub-lethal concentrations. Conditions were chosen that ultimately could be considered to exert both a lethal and a sub-lethal challenge to the population of cells. The MIC (as defined by the Clinical and Laboratory Standards Institute) itself is not an appropriate metric of concentrations which are inhibitory or sub-inhibitory in the context of our assay, as exposure of the antibiotics to cells occurs during logarithmic growth (approximately 10^7^–10^8^ cfu/mL) as opposed to exposure upon inoculation (∼10^5^ cfu/mL). However, we have found that the CLSI defined MIC is a convenient metric to reference to define inhibitory vs sub-inhibitory concentrations for most of the antibiotics examined here. As a general rule we observed growth modulation to mid-logarithmic cultures at concentrations of antibiotics that were 10-fold larger than their associated MIC (10×MIC), whereas concentrations approximating the MIC (1×MIC) were insufficient to impede mid-logarithmic cultures. This working definition is also comparable to concentrations chosen by other researchers in similar assays.[34] The growth rate of cells exposed to 1×MIC concentrations of fosmidomycin, kanamycin, or ampicillin were not statistically different than an untreated control over 60 min, whereas higher concentrations modulated the growth rate more significantly over this same time (Figure 2).

Quantitative RT-PCR relies upon analysis of transcription of genes of interest relative to an internal control that is expressed stably under the conditions examined. Selection of appropriate housekeeping genes in bacterial systems is not trivial for a number of reasons, including the variance of gene expression in various bacterial growth stages.[48] Previous transcriptional examinations of *Salmonella* species have used genes encoding 16S RNA or *gmk* as internal standards,[49] however, the expression of these genes have been reported to vary under certain dichotomous conditions (e.g. comparisons of bacteria in exponential vs stationary growth),[48] and thus must be carefully selected. In this study, we selected the *oraA* gene as an internal control. The *oraA* gene (also known as *recX*) encodes a protein that interacts with and regulates the activity of RecA during homologous recombination initiated as part of the DNA damage inducible SOS response. Despite its regulation in the DNA damage stress response, previous studies have shown that *oraA* is stably expressed in *E. coli* upon exposure to various sub-inhibitory concentrations of antibiotics (including ampicillin, kanamycin, rifampicin, norfloxacin, and fosmidomycin) over time [32], [34]. As these conditions were very similar to those we wished to examine in *Salmonella*, we rationalized that *oraA* could be a reliable housekeeping gene for our studies. Under certain conditions, *oraA* is subject to regulation, particularly those that result in significant DNA damage such as high concentrations of fluoroquinolones [34]. In general, gene expression patterns in any antibiotic exposure experiment are influenced not only by the action of the antibiotic with its intracellular target, but also from secondary metabolic and cellular affects associated with a decline in cellular health/viability from disruption of the targeted (requisite) pathway. As we wished to minimize the influence of secondary effects on transcription in our studies, the use of *oraA* was more suitable in than other standard housekeeping genes. First, *oraA* served as a housekeeping gene under conditions of mild antibiotic stress. Second, since *oraA* expression is induced under concentrations of antibiotics that elicit pronounced secondary effects (such as DNA damage), its stable expression itself reaffirmed that any influences to the global transcriptional response by secondary effects were minimal. We observed *oraA* to be stably expressed over various antibiotic concentrations, and furthermore, our probe for *oraA* is efficiently amplified over five orders of magnitude of RNA concentration (Figure S2). Under the conditions examined here, the expression of *oraA* was comparable to the expression of *gapA*, which is also commonly used as a bacterial housekeeping gene.[27], [49] Although the *oraA* probe proved useful for the sub-inhibitory antibiotic exposure experiments described here, caution must be taken when considering *oraA* as a housekeeping gene for other studies.

**Figure 3:**
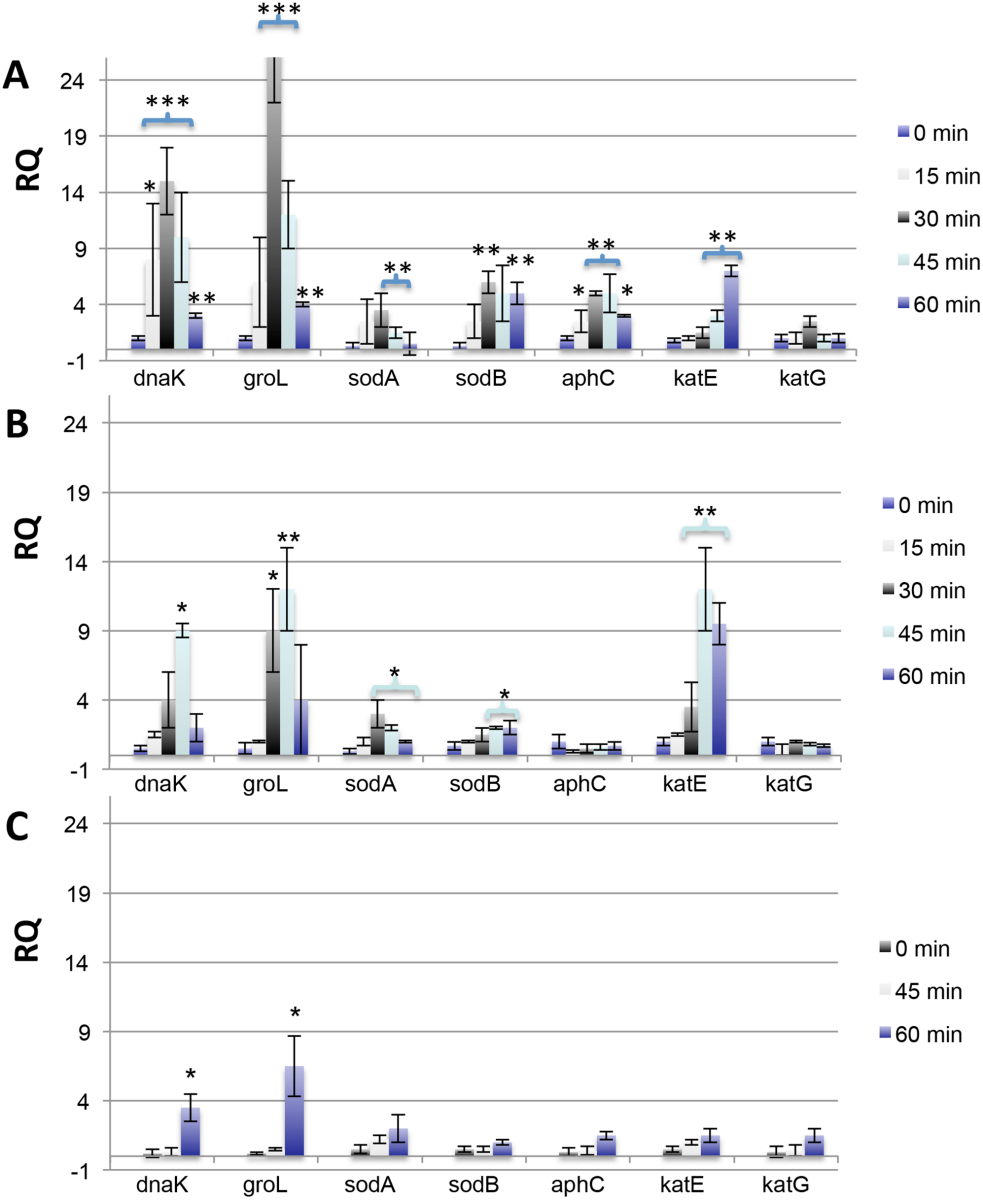
Time course of gene regulation for exposures to fosmidomycin, kanamycin, and tetracycline. Gene expression (in relative quantitation, or RQ, compared to untreated controls) is presented for cells challenged with the following antibiotic concentrations: a) fosmidomycin (20 **µ**g/mL), b) kanamycin (2 **µ**g/mL) and c) tetracycline (0.25 **µ**g/mL). All experiments were performed on three independent replicates and errors presented as the standard error of the mean (* = p<0.05, ** = p<0.01, *** = p<0.001).

Using *oraA* as an internal standard, the transcription of a panel of seven genes implicated in the response of cells to oxidative stress was undertaken at 15 min intervals over 1 h. Furthermore, RNA was isolated directly from cells immediately after antibiotic application as a 0 min time point. The genes examined in this panel included catalases (*katE*, *katG*), superoxide dismutases (*sodA*, *sodB*), an alkylhydrogen peroxide reductase (*ahpC*), and two genes associated with general stress responses in bacteria (*dnaK*, *groL*). Upon exposure to 20 **µ**g/mL fosmidomycin (Figure 3a), both genes encoding superoxide mediators (*sodA*/*B*) and peroxide mediators (*ahpC*) were mildly upregulated after 30 min. Expression of other peroxide mediating genes (*katE*) followed at later time points. Upregulation of *dnaK* and *groL*, which are associated with a general stress response in many bacteria, including *S. typhimurium*, are noted throughout the timecourse and serve as a positive control that the cells are interacting with the administered antibiotic.[50] In kanamycin exposed cells, upregulation of *katE* was similarly observable (Figure 3b), but the genes encoding the superoxide dismutases, *aphC* or *katG* were less clearly regulated at the chosen time intervals. Sub-lethal ampicillin exposures resulted in no statistically relevant regulation of *katG*/*aphC*, although some regulation of *katE* was seen (Figure S3). Virtually no regulation outside of the *groL*/*dnaK* genes was observed for cells proffered the bacteriostat tetracycline (Figure 3c). While the timing and fold induction of each of the genes in the expression profiles are not identical among the examined antibiotics, what is clear is that all of the genes in every exposure are upregulated very mildly when compared to exposures with authentic oxidants. Liu and Imlay observe a 10–20-fold increase in *katG* and 50–100-fold increase in *aphC* when *E. coli* is exposed to 250 µM H_2_O_2_ for 60 min.[27] Similarly, we have observed an approximately 50-fold increase in *sodA* and 75-fold increase in *soxS* after 45 min of exposure of *S. typhimurium* to nanoparticle-encapsulated silver carbene complexes (data not shown), and silver has been proposed to facilitate cell killing in part through its oxidative reactivity.[47], [51] The redox-cycling antibiotic paraquat has been shown to dramatically upregulate *soxS* when applied to *E. coli*.[52].

### Fosmidomycin Challenged Cells are Sensitized to H_2_O_2_

**Figure 4:**
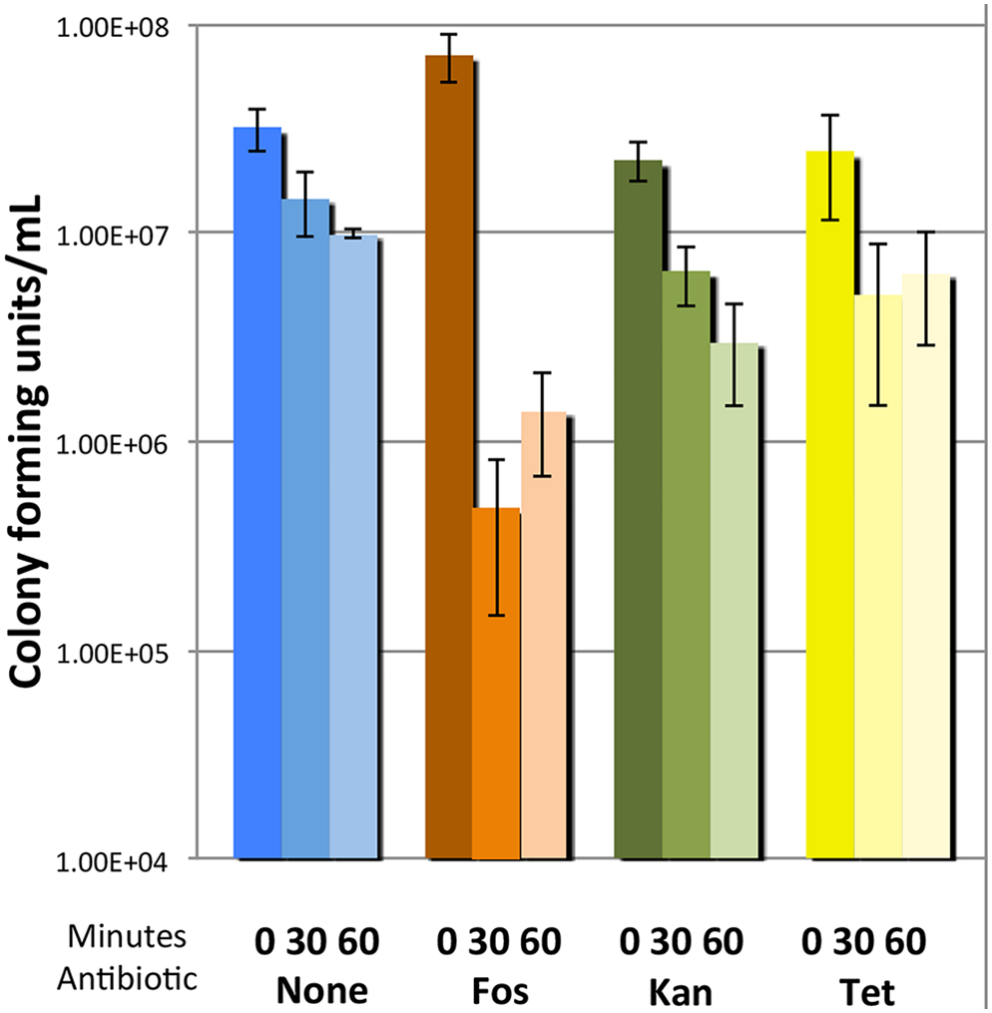
Cell viability of antibiotic challenged cells upon hydrogen peroxide exposure. Viable colony forming units/mL upon H_2_O_2_ (10 mM) treatment for untreated control cells, or cells previously challenged with fosmidomycin, kanamycin or tetracycline. The data represents the average of three independent tests and the error reflects the standard error of the mean.

The transcriptional experiments are consistent with our previous hypothesis that sub-inhibitory fosmidomycin exposure elicits oxidative stress in *S. typhimurium*. However, it is also well established that antibiotics at sub-inhibitory concentrations act not only as biocidal agents but also as intermicrobial signals.[33] Furthermore, the presence of redox-cycling antibiotics themselves, as opposed to the intracellular ROS they promote, is known to affect upregulation of the SoxS regulon.[53] It is possible that exposure to dilute fosmidomycin signaled the expression and accumulation of ROS mediation enzymes through hormesis. In this case, exposure to fosmidomycin may ultimately result in cells that are more prepared to remediate (and thus, more resistant to) ROS than their unexposed counterparts. The transcriptional and translational data alone is insufficient for us to conclude whether fosmidomycin exposed cells are experiencing oxidative stress or are preemptively accumulating cellular defenses against oxidation through a hormetic response. In order to examine the effect of fosmidomycin exposure on the cellular oxidative stress defense, we exposed *S. typhimurium* that had been challenged with fosmidomycin (200 **µ**g/mL) for 45 min to 10 mM H_2_O_2_ and compared the rate of viable cell depletion over time to an unchallenged control. Similarly, we also examined cells exposed to kanamycin (20 **µ**g/mL) and tetracycline (2.5 **µ**g/mL) (Figure 4). In this case, the concentrations chosen reflect a roughly ten fold increase over the MIC for each antibiotic, in order to accentuate any secondary effects elicited by the compounds. After 30 min, the fosmidomycin exposed cells were killed by the H_2_O_2_ significantly more than unexposed cells or cells treated with kanamycin or tetracycline. Relatively little cell death was observed after 60 min total exposure in all of the treatments, which we attribute to some degree of desensitization of the cells to peroxide and some loss in potency of the peroxide in solution over the course of the assay. Both of these phenomena have been reported by others in similar assays.[27], [45].

## Conclusions

Many studies of emergent antibiotic resistance focus on the discrete mechanisms responsible for resistance, such as the incurrence of mutations to the antibiotic’s target or the presence/expression of a resistance marker. However, it has also been clearly demonstrated that sub-inhibitory exposures to antibiotics can elicit global changes in transcription,[11]–[13], [31] and ultimately these changes can also play an influential role in the adaptation of the cell to the antibiotic even without mutational events or the acquisition of a *de novo* resistance gene. As such, a transcriptional analysis of the changes elicited by dilute concentrations of antibiotics can provide valuable information to the community regarding the development of resistance in an erstwhile sensitive strain, and is particularly valuable when applied to novel antibiotic classes with little *a priori* information on discrete resistance mechanisms.

As described earlier, one interesting observation is that the concentrations of fosmidomycin examined here did not stimulate systems previously identified with fosmidomycin resistance, nor upregulate the MEP pathway itself. Numerous components of the electron transport chain were upregulated, and there were many genes regulated in a manner consistent with that expected for an organism experiencing oxidative stress. Aerobic organisms have developed elegant means for dealing with the reactivity of an oxygenic environment, including the sensing and detoxification of the ubiquitous byproducts of aerobic respiration, ROS [54]. Furthermore, bacteria generally have intracellular pools of many metabolites that can serve as antioxidants, and while these pools may not be considered to have a primary role in oxidant mediation per se, they ostensibly serve as an auxiliary barrier to intracellular oxidative damage[45]. Without question, the metabolic factors which underpin oxidative stress in aerobic organisms are complicated and not easily deciphered. As *S. typhimurium* has evolved to resist conditions within macrophages, the organism is equipped with numerous and redundant mechanisms for ROS mediation. Dedicated ROS sensing and mediation is controlled on a transcriptional level in *S. typhimurium* by the regulating elements SoxRS (*soxS* regulates numerous genes including *sodA*)[55] and OxyR (which regulates *katG* and *ahpCF*, among others).[56], [57] The alternative sigma factor **σ**s (RpoS) also regulates genes associated with ROS defense (e.g. *katE*).[58] In this work, we observe sub-inhibitory concentrations of fosmidomycin to result in a mild upregulation of genes associated with each of these regulatory networks relative to unchallenged cells. This is in contrast to observations with the other antibiotics examined here. The bactericidal agents kanamycin and ampicillin did elicit regulation of *katE* during the course of our qPCR assay, which is associated with RpoS, but we observed minimal regulation of OxyR or SoxRS related genes at the examined concentrations. Tetracycline (bacteriostat) did not significantly influence the regulation of genes associated with ROS mediation. However, as discussed previously, the extent of regulation we observed does not compare to those observed in cells that are exposed to H_2_O_2_ itself. It is unlikely that these concentrations of fosmidomycin (or other antibiotics) are affecting a large accumulation of intracellular ROS. It has been noted that a transcriptional response in these regulons is attributable to exposure to redox-cycling antibiotics themselves.[53] To our knowledge, no examination of fosmidomycin as a redox-cycling compound has been reported, however we expect the likelihood of the antibiotic to be able to act in this manner to be low. Unlike quinone-based antimicrobial agents, fosmidomycin does not have chemical functionalities that are able to transiently undergo oxidative and reductive processes in a facile manner. Furthermore, no turnover of fosmidomycin in the presence of NADPH is observed during *in vitro* assays of MEP synthase, which is observed for other redox-cycling compounds.[1], [2] Last, the upregulation of similar oxidative stress associated enzymes upon removal of ME from the culture medium of the CT12 strain, which effectively mimics the inhibition of the MEP pathway experienced by wild-type cells exposed to fosmidomycin in an antibacterial compound-independent manner, argues that the response we observe is due to inhibition of the MEP pathway itself. In any case, based on our peroxide-sensitivity assays, the fosmidomycin exposure does elevate the sensitivity of the cells to this compound, presumably because they are already experiencing oxidative stress as mediated by the action of the antibiotic.

**Figure 5:**
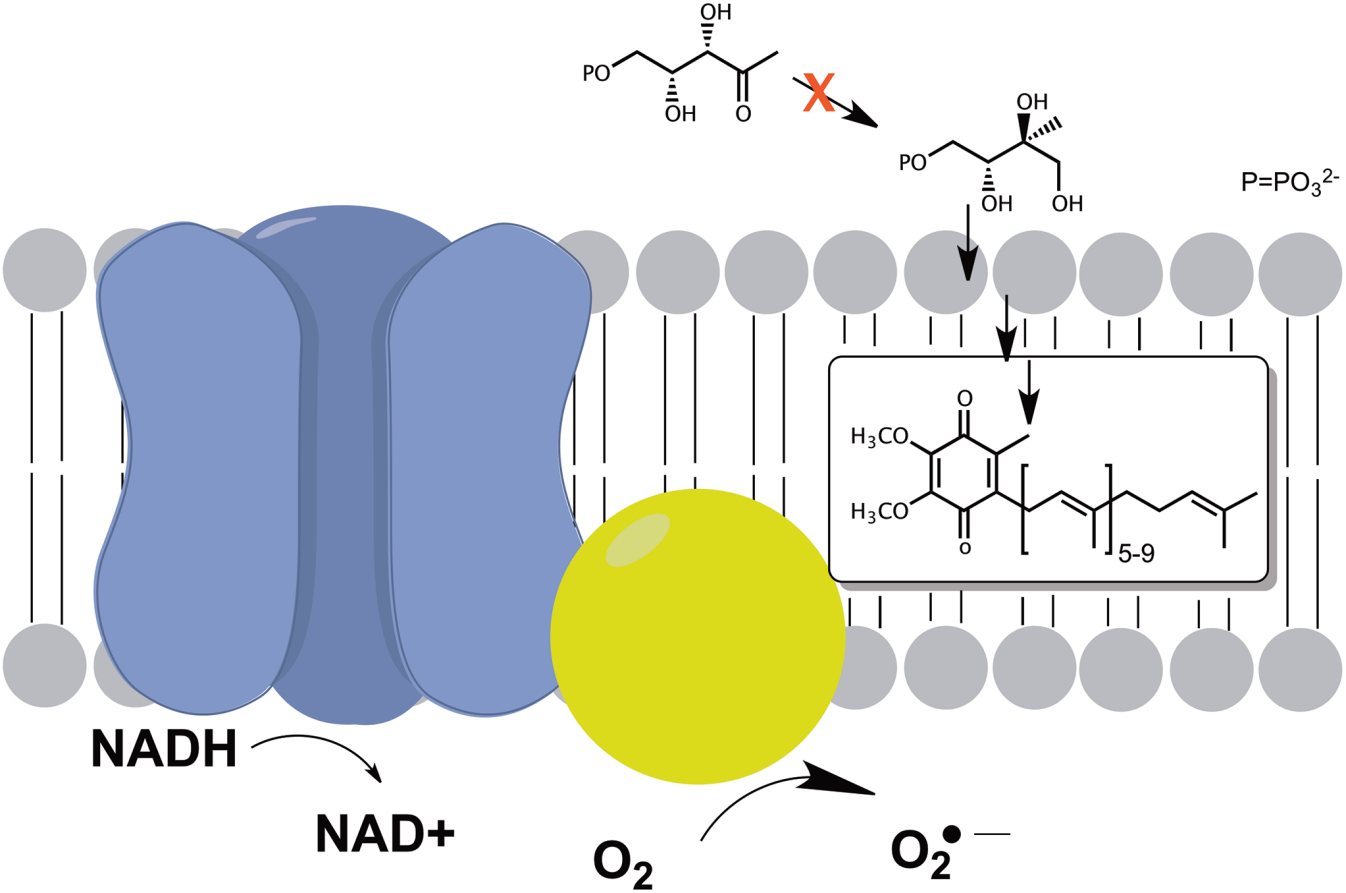
Potential Effects of Isoprenoid Deprivation on Cellular Respiration. Disruption of the MEP pathway reduces the cell’s ability to form late stage isoprenoid products such as ubiquinone and menaquinone. Impairment of bacterial respiration via inhibition of ubiquinone/menaquinone synthesis may decrease the cell’s ability to reduce oxygen into water, and ultimately may foster the production of reactive oxygen species through direct reduction of molecular oxygen.

At this time, the cellular factors that influence the fosmidomycin-mediated oxidative stress in *S. typhimurium* are not fully defined. However, given that the isoprenoid pathway in *S. typhimurium* largely exists to synthesize the prenyl side chain of ubiquinone and menaquinone, which are central to normal functioning of the electron transport chain, it is reasonable to postulate that a disruption in their concentration through inhibition of the MEP pathway would have a negative effect upon unfettered electron transport. Seaver and Imlay observed in vivo formation of H_2_O_2_ through the auto-oxidation of NADH dehydrogenase II (which normally serves to shuttle electrons from NADH to ubiquinone) in *E. coli* strains deficient in ubiquinone.[59] It is possible that a comparable phenomenon may be occurring in *S. typhimurium* cells that are impaired in their ability to make ubiquinone by fosmidomycin. (Figure 5). In the aforementioned work, intracellular ROS estimation was enabled by the use of a catalase/peroxidase deficient strain that released intracellular ROS into the surrounding medium (where it was subsequently quantified using commercial reagents). We have no access to a comparable strain of *S. typhimurium* deficient in ROS scavenging enzymes, and as such have been unable to estimate intracellular ROS after fosmidomycin exposure. The amount of ROS that originates from this process is believed to be a relatively small percentage of the total intracellular ROS in a cell, however (estimated to be produced at<1.5 **µ**M/s under average cellular respiratory conditions).[59] While this concentration of intracellular ROS is not reliably measurable in cells equipped with a full arsenal of ROS mediation mechanisms, it is sufficient to activate the OxyR transcriptional regulon (0.5–1.0 **µ**M intracellular ROS).[27] At this time it is unclear what effect the fosmidomycin exposures have on total ubiquinone/menaquinone concentration, or to what extent this may contribute to intracellular oxidative stress. Although degeneracy of the enzymatic activity displayed by MEP synthase has been observed in some strains, *S. typhimurium* is not proposed to be amongst them.[60] As such, fosmidomycin exposure is expected to have a direct effect on intracellular ubiquinone/menaquinone concentrations. A thorough investigation of the effect of MEP pathway inhibition on intracellular quinone concentrations is under current pursuit in our laboratory.

Inhibition of formation of late-stage products in the isoprenoid pathway may also be a minor contributor to the oxidative stress observed here. Many facultative aerobic bacteria, including *S. typhimurium*, produce small molecule antioxidants in addition to their enzymatic ROS mediating mechanisms. Turnbull and Surette have shown that aerobically grown *S. typhimurium* swarm cells overproduce the reductant glutathione relative to their aerobically grown swim cell counterparts, which is a contributing factor to the elevated antibiotic resistance observed in the swarm population.[46] Conversely, *Salmonella* strains deficient in cysteine biosynthesis (which is not used by the organism exclusively as a dedicated small molecule reductant, but certainly has a biologically relevant reduction potential) are inherently oxidatively stressed relative to a wild-type strain. Similar to many cysteine-based metabolites, products of the isoprenoid pathway are themselves very capable antioxidant compounds.[45] Although the main role of the isoprenoid pathway in *Salmonella* is not for antioxidants production per se, it is reasonable to expect that a strain deficient in the products of this pathway would also be intrinsically less protected from oxidation than a strain with robust isoprenoid production.

As discussed above, we believe the oxidative stress observed here is unlikely to be due to a marked increase in the intracellular ROS concentration of MEP pathway inhibited cells. Fosmidomycin exposed cells are not accumulating ROS to lethal concentrations as evidenced by the fact that the antibiotic does not cause appreciable cell death at the concentrations and exposure times examined here. However, our data does suggest that the judicious use of fosmidomycin may be viewed as a mechanism to cease cell growth and weaken endogenous oxidant defenses, which has been noted by others as a promising mechanism to potentiate antibiotics.[20], [28], [45] As such, the use of fosmidomycin (or other MEP inhibitors) in combination with synergistic antibiotics, similar to its use in treatments of the malaria parasite *Plasmodium falciparum*,[61]–[63] may also prove to be an advantageous antibacterial strategy.

## Materials and Methods

### General Methods and Reagents

A list of primers and probes used in qRT-PCR analysis is provided in the supplementary information (Table S1). Primers and probes were designed for all targets with PrimerExpress software (ABI) and were designed to afford amplicons of 100–150 nucleotides in length. *S. typhimurium* LT2 was purchased from the American Type Culture Collection (ATCC) and the mutant strain CT12 is an isoprenoid auxotroph derived from the LT2 strain. Briefly, CT12 is a derivative of strain RMC26 in which *dxr* was disrupted with a chloramphenicol acetyl transferase cassette as described for other MEP pathway genes [30]. All chemical reagents were purchased from Fisher or Sigma-Aldrich with the exception of fosmidomycin (Invitrogen) and methylerythritol (Echelon Biosciences). ME was used at a final concentration of 40 **µ**g/mL for all experiments.

### Antimicrobial Susceptibility Assays, Antibiotic Exposure Experiments and RNA Isolation

Minimal inhibitory concentrations for all examined antibiotics were determined using the broth microdilution method.[64] For antibiotic challenge experiments, a modified procedure was used. *S*. *typhimurium* cells were inoculated from a confluent culture into fresh LB (25 mL) and grown to an OD_600_ of 0.4 at 37°C with shaking. At this time they were exposed to each antibiotic. Sub-lethal/sub-inhibitory concentrations were examined for fosmidomycin (20 **µ**g/mL), kanamycin (2 **µ**g/mL), ampicillin (1 **µ**g/mL), and tetracycline (0.25 **µ**g/mL). In each case, antibiotics were added to the medium from a concentrated (1000X) stock solution to afford the final concentration of the compound. Cell viability over time under these concentrations was determined both spectrophotometrically (at OD_600_) and through enumeration of viable colony forming units via dilution and plating onto LB/agar at various times post-antibiotic exposure. Day-to-day variations in experimental preparations resulted in minor shifts of the cell densities, but sample-to-sample trends were consistent. For RNA isolation, aliquots (1–2 mL) were removed at each time point and subjected to centrifugation (12000 rpm, 1 min). After decanting the medium, the resulting bacterial pellet was washed briefly with 500 mL of PBS, overlaid with 100 mL of RNAlater (Ambion) and frozen on dry ice. The frozen aliquots were either processed directly or stored at −80°C for later analysis. RNA was isolated with a Qiagen RNEasy kit according to the manufacturers protocols and was immediately analyzed via qRT-PCR or cDNA microarray.

### Fosmidomycin-challenge and ME Deprivation Experiments for Differential Proteomics Analysis

Proteins were isolated for differential proteomics analysis from *S. typhimurium* CT12 cells, which had been deprived of ME relative to a control, or from wild-type *S. typhimurium* LT2 cells that had been challenged with fosmidomycin relative to a control. For ME deprivation experiments, *S. typhimurium* CT12 cells (10 mL) inoculated from a confluent culture were grown to mid-logarithmic phase (OD_600_ = 0.4) in LB medium supplemented with ME at 37°C with shaking. At this time the cells were centrifuged (4000 rpm, 15 min), the medium decanted, and the cell pellet was washed briefly with PBS (3 mL). The cell pellet was then resuspended in fresh LB (10 mL) and split into two equal volume aliquots, one of which was subsequently supplemented with ME. The cells were allowed to grow at 37°C with shaking for a further 45 min at which time they were isolated via centrifugation as described above. For fosmidomycin-challenge experiments, *S. typhimurium* LT2 cells (10 mL) were grown to mid-logarithmic phase (OD_600_ = 0.4) in LB medium at 37°C with shaking and subsequently split into two equal volume aliquots. One set of samples were subjected to a sub-lethal dose of fosmidomycin (10 **µ**g/mL) for 30 min, while the other was grown without antibiotic. All biological tests were performed in triplicate.

### Protein Isolation and Proteomic Analysis

Cells were lysed via sonication in extraction buffer (100 mM HEPES pH = 7.5, 1 mM EDTA, 0.5% Triton X-100, 2 M urea, 10 **µ**L/mL Sigma protease inhibitor cocktail). Protein isolates were either analyzed directly via SDS-PAGE or through tryptic digestion followed by labeling with isobaric isotope tags. For 1D SDS-PAGE, protein isolates were purified using a 4–12% Bis-Tris 1D SDS-PAGE gel (Life Technologies) at which time the proteins from each respective treatment were extracted and digested with Trypsin (Trypsin Gold; Promega) using the manufacturer’s suggested protocol. Briefly, gel lanes containing proteins for each respective treatment was cut into approximately 20 equal pieces, destained, dehydrated by immersing the pieces in 100% acetonitrile, and subsequently covered with elution buffer (50 mM Tris-HCl, 150 mM NaCl, and 0.1 mM EDTA; pH 7.5). The gel pieces were crushed and proteins extracted overnight at 30°C at which time they were then digested with trypsin (20 **µ**g/mL) at 37°C for 14–16 hours. For heavy isotope labeling studies, the protein isolates were digested with trypsin without purification and labeled with 4-plex iTRAQ reagents (AB SCIEX) as per the manufacturer’s protocol. Tryptic peptides from either test were subjected to strong cation exchange and C18 reversed-phase clean up and the clean peptide samples were lyophilized. Prior to MS, the lyophilized peptide samples were resuspended in 40 **µ**L of 0.1% formic acid. Each resuspended peptide mixture was spiked with 2 **µ**L of 400 fmol/**µ**L of MassPREP Yeast Alcohol Dehydrogenase as an external standard to monitor the consistency of the individual LC-MS/MS runs.

All nanoLC-MS/MS experiments were performed on a Waters Q-TOF Premier system equipped with nanoAcquity UPLC system (Waters Technology). Peptides were separated by reverse-phased LC and analyzed in both the full scan MS and in the collision-induced dissociation tandem mass spectrometry (MS/MS) modes. Briefly, 20 **µ**L of the peptide digest was loaded onto a C18 trapping column (Waters Symmetry C18 Trap Column, 5 **µ**m, 180 **µ**m×23 mm) followed by a 15 min wash with 5% (v/v) acetonitrile/0.1% (v/v) formic acid. Peptides were chromatographically separated on a BEH C18 nanoAcquity column (1.7 **µ**m, 100 **µ**m×100 mm) (Waters Technology) and eluted off the column using a linear gradient of increasing concentration of acetonitrile (in 0.1% (v/v) formic acid) as follows: 0–75 min, 5–50% (v/v) acetonitrile, 75–80 min, acetonitrile increased to 95% (v/v), 80–90 min acetonitrile maintained at 95%. The mass spectrometer was operated in the positive ion mode over 50–2000 m/z scan range and the ionization potential was set 2.1 kV. MS/MS data were acquired using a data-dependent acquisition format with the six most abundant ions from each MS scan further interrogated by MS/MS. Protein identification and iTRAQ ratios were calculated using PLGS 2.2.5 software (Waters Technology) using a minimum confidence score of 17. Database match was against the *S. typhimurium* LT2 NCBI protein sequence database. SDS-PAGE experiments were used to identify unique proteins to both respective treatments (ME deprivation/repletion) and for iTRAQ experiments, regulation was defined as variance of 20% or more in the isobaric label in identified peptides. All experiments were conducted with multiple replicates, including bacterial growth and exposure (3X), iTRAQ labeling (3X) and ESI sample analysis (3X).

### Microarray Analysis

RNA from all experimental samples were analyzed via microarray (MYcroarray, Inc., Ann Arbor, MI). RNA (200 ng) was converted to cDNA using the MessageAmp II Bacterial RNA Amplification kit (ABI), and amino-allyl UTP was incorporated into the transcripts for coupling with fluorescent dyes. Alexa Fluor 555 (Invitrogen) was coupled to the cDNA using the manufacturer’s protocols.

All cDNAs were hybridized to custom manufactured *S. typhimurium* microarrays. Each slide was composed of over 30,000 potential spots for probes, the majority of which (26,346) contain probes (45–47 mers) for *S. typhimurium* genes. The arrays also contained dedicated empty spots and positive controls for assessing hybridization/washing efficiency. Each gene was surveyed by one unique probe sequence, and six identical replicates of the probe are present on an individual slide. A total of 4,391 genes are analyzed in each array.

After fluorescent coupling and fragmentation, the resultant cDNA from three independent biological experiments was hybridized to arrays for 19 h at 50°C. The arrays were subsequently gently washed twice in SSPE buffer (150 mM NaCl, 1 mM EDTA, 6 mM NaH_2_PO_4_ and 4 mM Na_2_HPO_4_) for 3 min at 22°C, once for 3 min at 50°C, once for 3 min at 22°C, and once for 30 seconds at 22°C. After the final wash, the arrays were spun dry using a microarray centrifuge.

Following hybridization, the arrays were scanned in an Axon 4000B Scanner (Molecular Devices) and data was extracted from the scanned images using GenePix Pro Software (version 6.1.0.4). Each of the six probe replicates in an individual array was assigned a signal value based on the trimmed mean of the spots after background subtraction. Only genes displaying observable signal in four or more of the six replicates were included in the analysis of gene regulation. Fold change ratios were calculated for differentially expressed genes upon antibiotic administration relative to an untreated control, and statistical significance calculated using a Student’s t-test (two tails; two-sample equal variance).

Microarray raw data and normalized reads have been deposited and are publicly available at the Gene Expression Omnibus (GEO) website hosted by the National Center for Biotechnology Information (NCBI) (accession number GSE50480).

### qRT-PCR Analysis

Isolated RNA was either processed directly (qRT-PCR timecourse experiments) using a Superscript One-Step RT-PCR kit with Platinum Taq (Life Technologies) or first converted into cDNA (microarray validation experiments) using the High Capacity RNA to cDNA kit (ABI). Isolated RNA and synthesized cDNA were quantified spectrophotometrically at 260 and 280 nm.

RT-PCR for timecourse experiments was performed on an AB 7500 RT-PCR using dual-labeled gene specific probes (5′FAM-3′TAMRA) and primers specific to the gene of interest. In each case, 10 **µ**L of master mix was applied to 20 µM of each primer, and 5 **µ**M of the respective probe in a total volume of 20 **µ**L. After appropriate incubation to convert isolated mRNA to cDNA (30 min at 60°C), thermal cycling was performed in 40 cycles of 94°C (20 sec) and 62°C (60 sec). RT-PCR for microarray validation was performed either in the aforementioned manner or essentially as written without inclusion of gene specific probes using the SYBRgreen PCR Master Mix kit (Life Technologies) (Figure S4). Relative transcription was normalized to endogenous expression of the *oraA* gene and calculated using Voyager 7500 processing software, and statistical significance was calculated using a Student’s T-test (two tails; two-sample equal variance). In each case, melting curves for each amplicon was verified at the end of the experiment.

### Analysis of Bacterial Viability of Fosmidomycin-exposed Cultures via Fluorescence Spectroscopy

*S*. *typhimurium* cells were inoculated from a confluent culture into fresh LB (5 mL) and grown to an OD_600_ of 0.4 at 37°C with shaking, at which time the culture was exposed to fosmidomycin (20 **µ**g/mL) or grown with no antibiotic challenge. Aliquots of the cells were removed at various times post-exposure (0, 15, 30, 45, and 60 minutes), and prepared for fluorescent staining using procedures recommended by the manufacturer (BacLight LIVE/DEAD viability kit, Life Technologies). Briefly, aliquots (10 mL) were removed and diluted in cold buffered saline (100 mL) in a 96-well microplate at each time point to affect a final cell concentration of 10^7^ cfu/mL. At this time, the cells were mixed with SYTO 9 and propidium iodide (final concentrations of 6 and 30 **µ**M, respectively), and incubated in the dark for 15 minutes. Total cellular fluorescence was quantified at the appropriate excitation/emission maxima for each dye (480/500 nm for SYTO 9 and 490/635 nm for propidium iodide) using a microplate reader (BioTek). The percentage of viable cells in the fosmidomycin-exposed and untreated control cells was calculated by comparing the ratio of fluorescence associated with these tests to a calibration curve constructed of samples with varing percentages of live and heat/isopropanol killed cells. The biological tests, as well as the fluorescent readings in each of the assays, were performed in triplicate.

### Susceptibility with H_2_O_2_

*S*. *typhimurium* cells were inoculated from a confluent culture into fresh LB (5 mL) and grown to an OD_600_ of 0.4 at 37°C with shaking. At this time the culture was exposed to various antibiotics. For these experiments, higher concentrations of antibiotics were used relative to the sub-inhibitory challenge tests in order to maximize any effect toward exogenous oxidants elicited by the compounds. Antibiotics were added from 1000X stocks to afford the following final concentrations: fosmidomycin (200 **µ**g/mL), kanamycin (20 **µ**g/mL), and tetracycline (2.5 **µ**g/mL). Additionally, a sample of cells was allowed to grow without addition of antibiotic. After administration of antibiotic, the cultures were grown at 37°C with shaking for 30 min at which time the cells were collected by centrifugation, decanted and resuspended (OD_600_ of 0.4) in fresh LB containing 10 mM H_2_O_2_. At various time intervals, aliquots were harvested, the reaction terminated by the addition of catalase (2000 U/mL) and placed on ice. Cell viability over time in the presence of H_2_O_2_ was determined through enumeration of viable colony forming units via dilution and plating onto LB/agar. All biological tests were performed in triplicate.

## Supporting Information

Figure S1 **Cellular viability of bacterial suspensions after fosmidomycin exposure.** Cellular viability of fosmidomycin-exposed cells (blue) and untreated controls (red) was estimated from the total fluorescence of the samples. The ratio of the fluorescence associated with cells stained with SYTO 9 to those stained with either SYTO 9 or propidium iodide was used to estimate viability in the samples. All experiments were performed on three independent replicates and errors presented as the standard error of the mean.
(TIFF)

Figure S2 **Performance of the** ***oraA*** **probe in gene expression studies.** Amplification of the *oraA* probe measured as a) Ct relative to template concentration and b) **Δ**Rn relative to cycle for varying amounts of template RNA (100, 10, 1, 0.1 and 0.01 ng template in 50 **µ**L total). c) *gapA* (normalized to an *oraA* control) is stably expressed over the course of the assay for both fosmidomycin (20 **µ**g/mL) and kanamycin (2 **µ**g/mL) exposures. Observed expression values for a highly regulated gene (*groL*) are presented for comparison.
(TIFF)

Figure S3 **Time course of gene regulation for exposure to ampicillin.** Gene expression (in relative quantitation, or RQ, compared to untreated controls) is presented for cells challenged with ampicillin (1 **µ**g/mL). All experiments were performed on three independent replicates and errors presented as the standard error of the mean (* = p<0.05, ** = p<0.01, *** = p<0.001).
(TIFF)

Figure S4 **RT-PCR validation of selected regulated genes identified through microarray analysis.** Expression of a selected panel of genes was analyzed via qRT-PCR of total RNA isolated from cells exposed to fosmidomycin (20 **µ**g/mL) for 20 minutes.
(TIFF)

Table S1 **List of primers and probes used in this study.**
(DOCX)

Table S2 **Summary of regulated proteins observed upon 1D PAGE analysis of deprivation of ME from exponentially growing** ***S. typhimurium*** **CT12 cells relative to undeprived controls.**
(PDF)

Table S3 **Summary of regulated proteins observed upon iTRAQ analysis of fosmidomycin exposed** ***S. typhimurium*** **LT2 cells relative to untreated controls.**
(PDF)

Table S4 **Genes observed to be regulated by 50% or more upon fosmidomycin exposure relative to untreated controls.** Data is the result of three independent biological tests, each with eight technical replicates on the microarray. For comparison, a list of regulated genes upon similar exposure to kanamycin is provided.
(PDF)
