# Supplementary figures and images for: Sub-Inhibitory Fosmidomycin Exposures Elicits Oxidative Stress in Salmonella enterica Serovar typhimurium LT2
Source: PLoS One. 2014 Apr 21;9(4):e95271. doi: 10.1371/journal.pone.0095271 (PMC3994034; doi:10.1371/journal.pone.0095271)

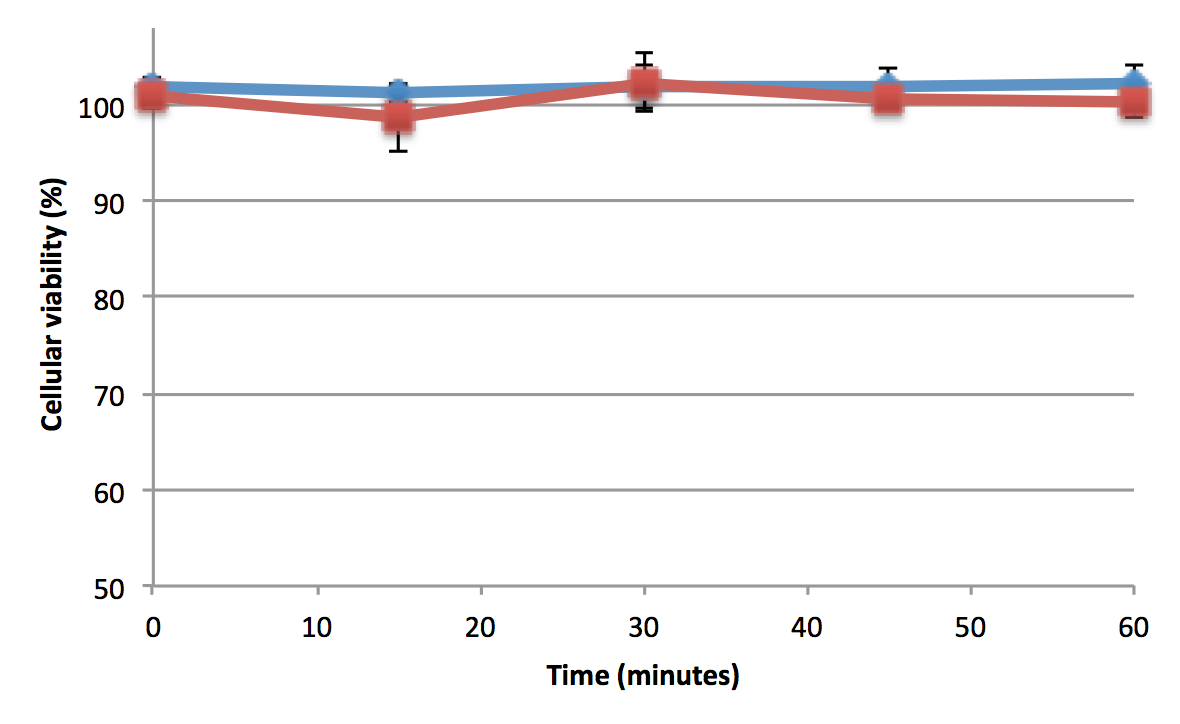

Supplement: Figure S1 — Cellular viability of bacterial suspensions after fosmidomycin exposure. Cellular viability of fosmidomycin-exposed cells (blue) and untreated controls (red) was estimated from the total fluorescence of the samples. The ratio of the fluorescence associated with cells stained with SYTO 9 to those stained with either SYTO 9 or propidium iodide was used to estimate viability in the samples. All experiments were performed on three independent replicates and errors presented as the standard error of the mean. (TIFF) [file pone.0095271.s001.tif]

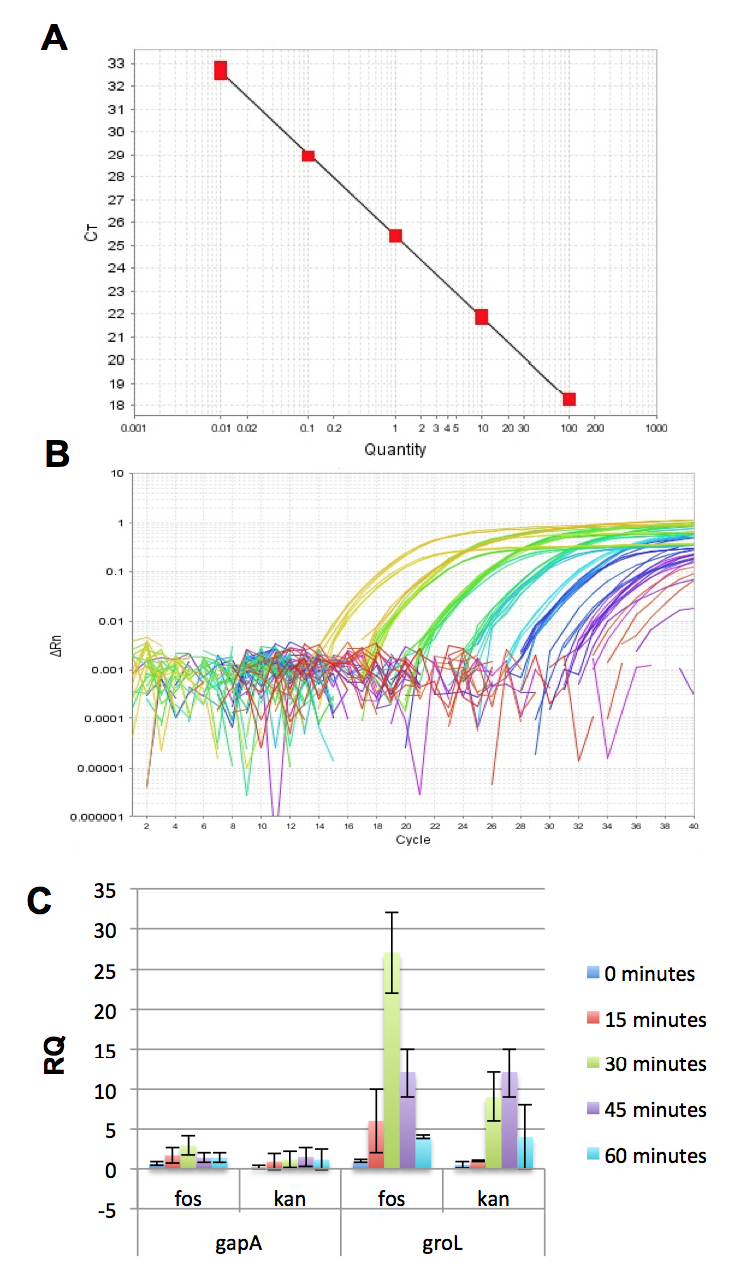

Supplement: Figure S2 — Performance of the oraA probe in gene expression studies. Amplification of the oraA probe measured as a) Ct relative to template concentration and b) ΔRn relative to cycle for varying amounts of template RNA (100, 10, 1, 0.1 and 0.01 ng template in 50 µL total). c) gapA (normalized to an oraA control) is stably expressed over the course of the assay for both fosmidomycin (20 µg/mL) and kanamycin (2 µg/mL) exposures. Observed expression values for a highly regulated gene (groL) are presented for comparison. (TIFF) [file pone.0095271.s002.tif]

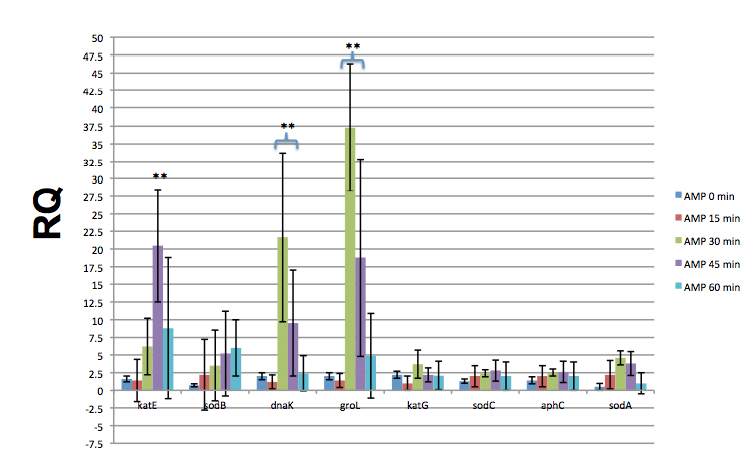

Supplement: Figure S3 — Time course of gene regulation for exposure to ampicillin. Gene expression (in relative quantitation, or RQ, compared to untreated controls) is presented for cells challenged with ampicillin (1 µg/mL). All experiments were performed on three independent replicates and errors presented as the standard error of the mean (* = p<0.05, ** = p<0.01, *** = p<0.001). (TIFF) [file pone.0095271.s003.tif]

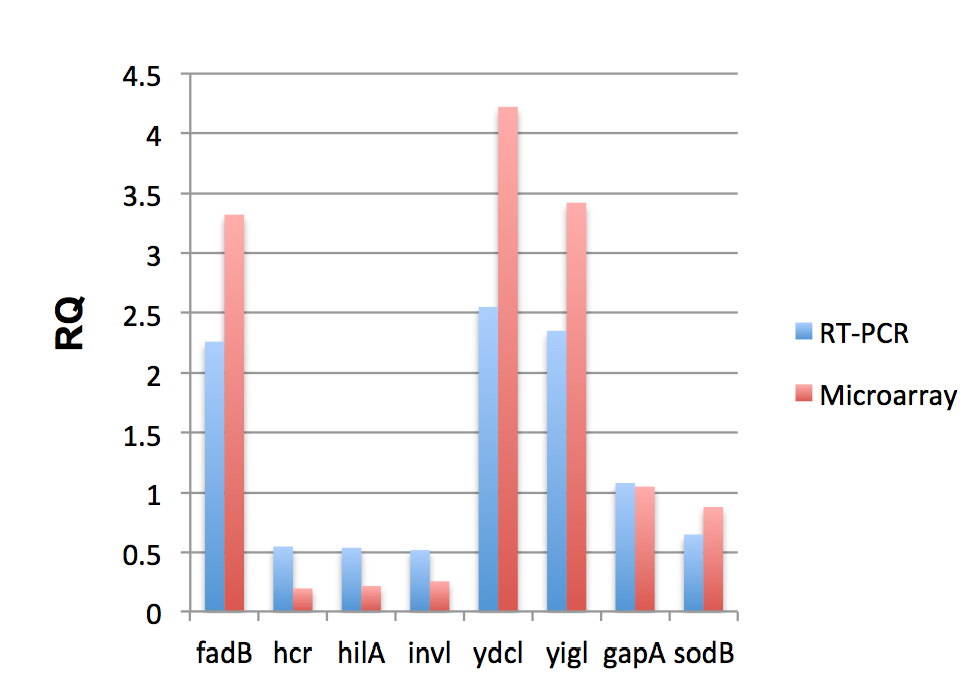

Supplement: Figure S4 — RT-PCR validation of selected regulated genes identified through microarray analysis. Expression of a selected panel of genes was analyzed via qRT-PCR of total RNA isolated from cells exposed to fosmidomycin (20 µg/mL) for 20 minutes. (TIFF) [file pone.0095271.s004.tif]
