# Supplementary material for: Sub-Inhibitory Fosmidomycin Exposures Elicits Oxidative Stress in Salmonella enterica Serovar typhimurium LT2
Source: PLoS One. 2014 Apr 21;9(4):e95271. doi: 10.1371/journal.pone.0095271 (PMC3994034; doi:10.1371/journal.pone.0095271)
Supplement: Table S1 — List of primers and probes used in this study. (DOCX) [file pone.0095271.s005.docx]

**Table S1: List of primers and probes used in this study.**

Target gene Sequence

*ahpC*

Forward 5’-GAAGCTGGGCGTAGACGTTT-3’

Reverse 5’-AGCTGCTGTGCCATGCTTT-3’

Probe 5’-FAM-TCCGTCTCAACCGATACTCACTTCACGC-TAMSp-3’

*dnaK*

Forward 5’-TGACTCCGCTTATCACCAAAAA-3’

Reverse 5’-TGGTTGTCTTCCGCAGTAGAGA-3’

Probe 5’-FAM-ACCACCATCCCGACCAAGCACAG-TAMSp-3’

*fadB*

Forward 5’- GAGGAAGATACCGACCAGCG-3’

Reverse 5’- AAAGGCATGGTCGCACAAAC-3’

*groL*

Forward 5’-AGGCGGCGACGGTAACTAC-3’

Reverse 5’-CCAGGATACCCATATCGATCATG-3’

Probe 5’-FAM-TTACAACGCAGCAACTGAAGAATACGGCA-TAMSp-3’

*hilA*

Forward 5’- TATCGCAGTATGCGCCCTTT-3’

Reverse 5’- CAAGAGAGAAGCGGGTTGGT-3’

*hcr*

Forward 5’- CGTACCGATCGGGGCATAAA-3’

Reverse 5’- ACGCTAGTGGCGGAAAATCA-3’

*invI*

Forward 5’- CGGCCTCTTCCTGCTGTATC-3’

Reverse 5’- CAGACAGCTCAGTCGTGAGG-3’

*katE*

Forward 5’-TGCATTGATCAGACGGAAGGT-3’

Reverse 5’-GCGGTATTCCACGCAGTTATC-3’

Probe 5’-TAM-TGAATGCCGAACCCTTCCATCGTC-TAMSp-3’

*katG*

Forward 5’-GCGGGTCGTGGTCAACAG-3’

Reverse 5’-GCCTTATCCAGGCTGACGTTAT-3’

Probe 5’-FAM-TTGCGCCGCTTAACTCCTGGCC-TAMSp-3’

*oraA*

Forward 5’-TGGCACCAGGCGATCAC-3’

Reverse 5’-AATGGGCCGGAAGAGATTG-3’

Probe 5’-FAM-CGTTCATAATCGTCTGCCGTCGCA-TAMSp-3’

*sodA*

Forward 5’-TTCTGGAAAGGGCTGAAAAAAG-3’

Reverse 5’-CCGAAGTCACGCTCGATAGC-3’

Probe 5’-FAM-CACCACTCTGCAGGGCGATCTGAAA-TAMSp-3’

*sodB*

Forward 5’-TTTGCCATCAGCGCTTTTTA-3’

Reverse 5’-CCGATGCCGCTATCAAAAAC-3’

Probe 5’-FAM-CAGCCATGTCCATCCGGAACCAA-TAMSp-3’

*ydcI*

Forward 5’- ATACATTTGTCGCCGTTGCG-3’

Reverse 5’- AGCGTCCTCTTTGCGATTCA-3’

*yigI*

Forward 5’- CGACGGCCACTTTATTCCCT-3’

Reverse 5’- AACTGGGCGCAAAGCATTTT-3’
