# Supplementary material for: Sub-Inhibitory Fosmidomycin Exposures Elicits Oxidative Stress in Salmonella enterica Serovar typhimurium LT2
Source: PLoS One. 2014 Apr 21;9(4):e95271. doi: 10.1371/journal.pone.0095271 (PMC3994034; doi:10.1371/journal.pone.0095271)
Supplement: Table S2 — Summary of regulated proteins observed upon 1D PAGE analysis of deprivation of ME from exponentially growing S. typhimurium CT12 cells relative to undeprived controls. (PDF) [file pone.0095271.s006.pdf]

Table S2: Summary of regulated proteins observed upon 1D PAGE analysis of deprivation of ME to exponentially growing CT12.

Unique proteins observed upon ME deprivation

protein mw	accession number	percent Coverage	# Unique Peptides	Score	Protein
65567.4	16420277	14	5	56.97	oligopeptide transport protein with chaperone properties
25978.1	16423136	24	5	55.7	purine-nucleoside phosphorylase
24249	16421901	22	3	47.6	stringent starvation protein A
20545.7	16422705	13	3	44.43	component in transcription antitermination
31294.2	16421031	17	3	42.71	dihydrodipicolinate synthase
26916.9	16422647	15	3	40.21	triosephosphate isomerase
9312.5	16422655	51	3	38.7	putative cytoplasmic protein
17609	16421033	22	2	36.64	thioredoxin dependent thiol peroxidase
12907.9	16419953	21	2	31.76	putative glutaredoxin protein
19524.4	16420877	17	2	31.71	putative cytoplasmic protein
82322.7	16421013	4	2	31.66	putative transferase
37545	16422925	13	2	30.04	component of modulator for protease specific for FtsH phage lambda cII repressor
53942.1	16422477	5	2	29.98	ketol-acid reductoisomerase
16687.4	16421940	18	2	29.58	acetylCoA carboxylase, BCCP subunit
6482.6	16420340	42	2	28.78	putative cytoplasmic protein
21308	16419951	12	2	27.6	iron superoxide dismutase
61634.4	16422416	6	2	27.45	putative preprotein translocase subunit YidC
11085.6	16421786	25	2	26.83	putative inner membrane protein
65492.4	16422905	6	2	26.19	fumarate reductase
19279.8	16420472	13	2	25.51	cytoplasmic ferritin
6446.4	16419707	45	2	24.61	50S ribosomal subunit protein L32
45414.5	16420493	9	2	23.59	N-methylation of lysine residues in flagellin
23525.3	16422677	9	2	23.19	putative transaldolase
36799.1	16422978	11	2	22.88	fructose-bisphosphatase
47326.5	16421907	5	2	22.21	serine endoprotease
19470.1	16422045	15	2	22.07	shikimate kinase I
12014.9	16418993	18	1	21.6	putative cytoplasmic protein
29102.5	16422259	11	2	20.9	putative transcriptional GntR family regulator for lct operon
51387.5	16420424	7	2	20.15	pyruvate kinase II
33048.5	16420986	10	2	20.08	putative iron-dependent peroxidase
29435.8	16418750	11	2	19.65	putative outer membrane lipoprotein
76274.2	16420690	3	2	19.52	methionine tRNA synthetase
17656.1	16421857	12	2	19.5	transcription elongation factor
55950.2	16419120	4	1	18.84	alkyl hydroperoxide reductase, F52a subunit
51683.1	16423156	10	2	18.53	sensory kinase (alternative) in two-component regulatory system with CreB (or alternatively PhoB)
22574.3	16422250	8	1	18.29	putative glutathione S-transferase

19074.2	16422814	7	1	18.05	ssDNA-binding protein
18856.9	16419471	9	1	18.02	regulator for lrp regulon and high-affinity branched-chain amino acid transport system
13269.4	16419722	10	1	17.72	putative protein kinase C inhibitor
20623.4	16422896	6	1	17.7	elongation factor P
22810.4	16418952	5	1	17.65	putative lipoprotein
22903.5	16421885	10	1	17.52	sigma cross-reacting protein 27A (SCRP-27A)
37944.5	16421374	5	1	17.21	DNA strand exchange and recombination protein
92049.7	16422929	4	2	17.06	putative exoribonuclease

Unique proteins no longer observed upon ME deprivation

protein mw	accession number	percent Coverage	# Unique Peptides	Score	Protein
47705.1	16420935	16	4	48.25	transport of long-chain fatty acids
33215.9	16420909	15	3	39.91	acetylCoA carboxylase, beta subunit
20867.7	16419635	14	2	30.17	trp-repressor binding protein
45562	16423031	11	3	23.77	putative arginine deiminase
25089.1	16420868	15	2	26.69	NADH dehydrogenase I chain B
42285.4	16422500	11	2	25.33	uroporphyrinogen III methylase
69862	16419079	6	2	23.02	outer membrane N-acetyl phenylalanine beta-naphthyl ester-cleaving esterase
12806	16422714	19	2	21.39	putative cytoplasmic protein
88695.4	16418495	5	2	20.93	aspartokinase I
58388.5	16420151	7	2	20.24	methyl-accepting chemotaxis protein III
75187.4	16419547	5	2	19.9	Gifsy-2 prophage Clp protease-like protein
37267.6	16422988	12	2	19.36	putative dehydrogenase
26978.4	16419045	21	2	19.32	UDP-2,3-diacylglucosamine hydrolase
92083.7	16422678	2	2	19.3	general PTS family enzyme I
55921.8	16420422	5	2	19.19	glucose-6-phosphate dehydrogenase
84299.2	16422181	4	2	18.88	putative cellulose synthase
36851.2	16420505	7	2	18.56	flagellar biosynthesis protein
39658.1	16418815	11	2	18.02	DNA polymerase IV
24910.7	16422961	23	2	17.93	putative cell morphogenesis
35778.4	16421129	5	1	17.68	leader peptidase (signal peptidase I), serine protease
133812.9	16421543	3	2	17.24	exonuclease V, beta chain
