# Supplementary material for: Sub-Inhibitory Fosmidomycin Exposures Elicits Oxidative Stress in Salmonella enterica Serovar typhimurium LT2
Source: PLoS One. 2014 Apr 21;9(4):e95271. doi: 10.1371/journal.pone.0095271 (PMC3994034; doi:10.1371/journal.pone.0095271)
Supplement: Table S3 — Summary of regulated proteins observed upon iTRAQ analysis of fosmidomycin exposed S. typhimurium LT2 cells relative to untreated controls. (PDF) [file pone.0095271.s007.pdf]

Upregulated proteins upon exposure to fosmidomycin

Accession	Description	MW	pI	Peptides	Coverage
AAL19402.1	peptidyl prolyl cis trans isomerase	48036	4.6436	6	14.3519
AAL19907.1	pyruvate formate lyase I induced anaerobically	84950	5.671	8	12.8947
AAL20003.1	putative membrane component hydrogenase	37491	5.499	5	11.7143
AAL20301.1	murein lipoprotein	8386	9.8229	1	15.3846
AAL20752.1	cold shock protein	7397	7.7023	2	26.087
AAL21169.1	outer membrane protein 1b ib c	41213	4.4396	1	1.8519
AAL21453.1	lysine decarboxylase 1	81182	5.9795	8	12.605
AAL21540.1	putative formate acetyltransferase	14335	4.9156	4	42.5197
AAL21832.1	enolase	45570	5.0839	6	15.7407
AAL22228.1	malate dehydrogenase	32455	5.9676	6	15.0641
AAL22278.1	RNA polymerase alpha subunit	36489	4.7803	5	16.7173
AAL22286.1	30S ribosomal subunit protein S5	17592	10.5595	4	24.5509
AAL22295.1	50S ribosomal subunit protein L29	7255	10.5154	4	58.7302
AAL22304.1	30S ribosomal subunit protein S10	11759	10.3264	2	25.2427
AAL22308.1	protein chain elongation factor EF Tu	43256	5.1462	17	40.3553
AAL23153.1	chaperone Hsp60 with peptide dependent ATPase activity	57249	4.6483	8	14.781
AAL19559.1	alkyl hydroperoxide reductase C22 subunit	20734	4.8483	2	12.2995
AAL19684.1	cytochrome d terminal oxidase polypeptide subunit I	58277	6.4471	2	1.7241

Downregulated proteins upon exposure to fosmidomycin

Accession	Description	MW	pI	Peptides	Coverage
AAL19189.1	histone like protein located in outer membrane	17894	10.2534	1	9.9379
AAL20669.1	DNA binding protein HLP II	15532	5.0903	2	10.219
AAL21127.1	50S ribosomal subunit protein L25	10534	9.9401	2	24.4681
AAL21359.1	putative detox protein in ethanolamine utilization	9836	6.0899	1	14.5833
AAL21565.1	30S ribosomal subunit protein S16	9229	11.0544	4	43.9024
