# Supplementary material for: Sub-Inhibitory Fosmidomycin Exposures Elicits Oxidative Stress in Salmonella enterica Serovar typhimurium LT2
Source: PLoS One. 2014 Apr 21;9(4):e95271. doi: 10.1371/journal.pone.0095271 (PMC3994034; doi:10.1371/journal.pone.0095271)
Supplement: Table S4 — Genes observed to be regulated by 50% or more upon fosmidomycin exposure relative to untreated controls. Data is the result of three independent biological tests, each with eight technical replicates on the microarray. For comparison, a list of regulated genes upon similar exposure to kanamycin is provided. (PDF) [file pone.0095271.s008.pdf]

Table S4: Genes observed to be regulated by 50% or more upon fosmidomycin exposure. Data is the result of three independent biological tests, each with eight technical replicates on the microarray. For comparison, a list of regulated genes upon similar exposure to kanamycin is provided.

Genes upregulated by 50% or more upon fosmidomycin exposure (p<0.05)

			Fosmidomycin challe		Kanamycin challenge		
**Name**	**Gene Name**	**Predicted Gene Product**	**p value**	**Fold change upon exposure**	**p value**	**Fold change upon exposure**	**Putative Role**
STM2342	sgaT	PTS system ascorbate-specific transporter	0.001	4.79	0.022	2.35	Carbohydrate transport/phosphorlyation
STM2343	null	putative cytoplasmic protein	0.001	4.29	0.009	2.38	Unknown
STM1630	null	putative inner membrane protein	0.025	4.22	0.256	3.83	Cell wall components/synthesis
STM1625	ydcI	putative transcriptional regulator	0.005	4.22	0.007	5.68	DNA transcription
STM0443	cyoA	cytochrome o ubiquinol oxidase subunit II	0.003	4.10	0.000	4.59	Electron transport
STM0442	cyoB	cytochrome o ubiquinol oxidase subunit I	0.003	3.93	0.001	3.58	Electron transport
STM4265	soxS	DNA-binding transcriptional regulator SoxS	0.001	3.51	0.955	0.99	ROS response
STM3956	yigI	hypothetical protein	0.006	3.42	0.007	4.53	Unknown
STM2340	null	putative transketolase	0.001	3.33	0.002	2.20	Carbohydrate utilization
STM3983	fadB	multifunctional fatty acid oxidation complex	0.018	3.32	0.042	3.46	Fatty acid catabolism
STM0367	prpR	prp operon regulator	0.028	3.24	0.403	1.32	DNA transcription
STM2341	null	putative transketolase	0.005	3.23	0.026	2.21	Carbohydrate utilization
STM4055	sodA	superoxide dismutase	0.007	3.02	0.004	6.78	ROS response
STM2355	argT	lysine/arginine/ornithine transport protein	0.008	2.94	0.011	2.47	Amino acid transport
STM4165	rsd	anti-RNA polymerase sigma 70 factor	0.006	2.92	0.016	2.35	DNA transcription
STM2282	glpQ	glycerophosphodiester phosphodiesterase	0.030	2.81	0.799	0.94	Fatty acid catabolism
STM0309	fadE	acyl-CoA dehydrogenase	0.007	2.59			Fatty acid catabolism
STM0664	gltJ	glutamate/aspartate transporter	0.012	2.59	0.019	2.17	Amino acid transport
STM2785	tctD	regulatory protein	0.013	2.53	0.179	1.69	DNA transcription
STM2993	recD	exonuclease V subunit alpha	0.030	2.53	0.020	2.39	DNA repair
STM1802	dadX	alanine racemase	0.019	2.50	0.016	3.06	Amino acid metabolism
STM0665	gltI	glutamate and aspartate transporter subunit	0.008	2.50	0.043	1.94	Amino acid transport
STM4275	acs	acetyl-CoA synthetase	0.002	2.49	0.024	2.35	Fatty acid metabolism
STM1125	putP	major sodium/proline symporter	0.003	2.29	0.004	2.07	Amino acid transport
STM1803	dadA	D-amino acid dehydrogenase small subunit	0.023	2.25	0.014	2.86	Amino acid metabolism
STM4086	glpK	glycerol kinase	0.035	2.25	0.704	0.92	Carbohydrate utilization
STM3521	null	putative ribonucleoprotein related-protein	0.007	2.24			Unknown
STM0663	gltK	glutamate/aspartate transporter	0.024	2.24	0.040	1.99	Amino acid transport
STM4274	yjcH	putative inner membrane protein	0.007	2.21	0.009	2.22	Cell wall components/synthesis
STM2344	null	putative phosphotransferase system enzyme II A	0.025	2.14	0.125	1.66	Cell signaling
STM2784	tctE	regulatory protein	0.028	2.14	0.069	1.56	DNA transcription
STM4160	thiG	thiazole synthase	0.031	2.13	0.155	1.65	Cofactor biosynthesis
STM3877	asnA	asparagine synthetase AsnA	0.032	2.10	0.202	0.72	Amino acid biosynthesis
STM1304	astA	arginine succinyltransferase	0.009	2.08	0.011	2.53	Amino acid catabolism
STM3982	fadA	3-ketoacyl-CoA thiolase	0.050	2.07	0.031	2.40	Fatty acid catabolism
STM2789	null	hypothetical protein	0.011	2.06	0.005	2.83	Unknown

STM2352	hisM	histidine/lysine/arginine/ornithine transport	0.031	2.05	0.213	1.39	Amino acid transport
STM4162	thiF	thiamine biosynthesis protein ThiF	0.009	2.04	0.018	1.88	Cofactor biosynthesis
STM4398	cycA	D-alanine/D-serine/glycine permease	0.007	2.04	0.037	1.71	Amino acid transport
STM2388	fadJ	multifunctional fatty acid oxidation complex	0.025	2.02	0.015	1.92	Fatty acid catabolism
STM3457	kefB	glutathione-regulated potassium-efflux system	0.023	2.00	0.074	1.72	Transport
STM3158	exbD	biopolymer transport protein ExbD	0.013	1.99	0.041	3.00	Transport
STM4126	udhA	soluble pyridine nucleotide transhydrogenase	0.006	1.98	0.018	1.71	Oxidoreductase
STM4184	aceA	isocitrate lyase	0.043	1.92	0.002	4.06	Carbohydrate utlilzation
STM2188	mglC	beta-methylgalactoside transporter inner	0.027	1.92	0.049	1.51	Carbohydrate transport
STM4163	thiE	thiamine-phosphate pyrophosphorylase	0.026	1.91			Cofactor biosynthesis
STM4329	groES	co-chaperonin GroES	0.001	1.89	0.003	2.08	Protein folding
STM1795	null	putative glutamic dehyrogenase-like protein	0.002	1.89	0.002	1.90	Amino acid catabolism/metabolism
STM0733	sdhD	succinate dehydrogenase cytochrome b556 small	0.021	1.85	0.006	1.80	Electron transport
STM1291	yeaA	methionine sulfoxide reductase B	0.003	1.82	0.023	2.01	Enzyme repair
STM2190	mglB	galactose transport protein	0.021	1.82	0.279	1.20	Carbohydrate transport
STM3339	nanA	N-acetylneuraminate lyase	0.015	1.82	0.017	2.06	Carbohydrate metabolism
STM0828	glnQ	glutamine ABC transporter ATP-binding protein	0.031	1.80	0.054	1.57	Amino acid transport
STM0441	cyoC	cytochrome o ubiquinol oxidase subunit III	0.004	1.78	0.061	1.44	Electron transport
STM4276	null	putative cytoplasmic protein	0.022	1.75	0.007	1.71	Unknown
STM4330	groEL	chaperonin GroEL	0.038	1.73	0.023	2.06	Protein folding
STM3019	yqeF	acetyl-CoA acetyltransferase	0.017	1.73	0.033	1.82	Acyltransferase
STM4037	fdoG	formate dehydrogenase alpha subunit	0.020	1.72	0.125	1.24	Organic acid utilization
STM3225	ygjU	serine/threonine transporter SstT	0.002	1.71	0.050	1.50	Amino acid transport
STM0440	cyoD	cytochrome o ubiquinol oxidase subunit IV	0.002	1.70	0.011	1.35	Electron transport
STM0662	gltL	glutamate/aspartate transporter	0.019	1.70	0.024	1.60	Amino acid transport
STM0734	sdhA	succinate dehydrogenase flavoprotein subunit	0.007	1.68	0.014	1.46	Electron transport
STM3688	null	putative cytoplasmic protein	0.024	1.68	0.090	1.55	Unknown
STM0012	dnaK	molecular chaperone DnaK	0.009	1.68	0.000	2.54	Protein folding
STM3159	exbB	biopolymer transport protein ExbB	0.027	1.68	0.020	2.97	Transport
STM0732	sdhC	succinate dehydrogenase cytochrome b556 large	0.008	1.67	0.010	1.51	Electron transport
STM0456	ybaE	putative ABC transporter periplasmic binding	0.041	1.66	0.008	1.45	Transport
STM3135	null	mannonate dehydratase	0.025	1.66	0.114	1.37	Carbohydrate utilization
STM0600	cstA	carbon starvation protein	0.006	1.64	0.079	1.30	Unknown
STM2354	hisJ	histidine transport protein	0.033	1.63	0.034	1.49	Amino acid transport
STM3830	dgoR	galactonate operon transcriptional repressor	0.038	1.62	0.078	1.40	DNA transcription
STM0830	glnH	glutamine ABC transporter periplasmic protein	0.027	1.61	0.125	1.30	Amino acid transport
STM1469	fumC	fumarate hydratase	0.032	1.61	0.100	1.36	Carbohydrate utilization/amino acid catabolism
STM1292	yeaC	putative cytoplasmic protein	0.002	1.61	0.000	1.81	Unknown
STM0829	glnP	glutamine ABC transporter permease protein	0.013	1.60	0.020	1.41	Amino acid transport
STM3363	yhcO	putative cytoplasmic protein	0.018	1.59	0.012	1.49	Unknown
STM4091	hslU	ATP-dependent protease ATP-binding subunit HslU	0.008	1.58	0.001	2.39	Protease
STM2437	yfeJ	glutamine amidotransferase	0.036	1.57	0.037	1.32	Amino acid catabolism/biosynthesis
STM0609	ahpF	alkyl hydroperoxide reductase F52a subunit	0.006	1.57	0.001	1.56	ROS response
STM0945	clpA	ATP-dependent Clp protease ATP-binding subunit	0.008	1.57	0.042	1.29	Protease
STM0439	cyoE	protoheme IX farnesyltransferase	0.023	1.56	0.194	1.23	Electron transport
STM3829	dgoK	2-oxo-3-deoxygalactonate kinase	0.038	1.56	0.005	1.59	Carbohydrate utilization

STM3700	gpsA	NAD(P)H-dependent glycerol-3-phosphate dehydro	0.022	1.54	0.148	1.38	Carbohydrate utilization
STM0928	nanH	neuraminidase	0.007	1.54	0.923	1.02	Cell wall components/synthesis
STM0226	lpxD	UDP-3-O-[3-hydroxymyristoyl] glucosamine	0.015	1.54	0.066	1.35	Cell wall components/synthesis
STM4408	msrA	methionine sulfoxide reductase A	0.042	1.53	0.010	1.95	Enzyme repair
STM4327	fxsA	FxsA	0.004	1.52	0.021	1.57	Cell wall components/synthesis
STM4233	ubiC	chorismate pyruvate lyase	0.004	1.51	0.177	1.22	Amino acid biosynthesis
STM0787	hutI	imidazolonepropionase	0.045	1.51	0.025	1.38	Amino acid degradation
STM0225	hlpA	periplasmic chaperone	0.030	1.51	0.168	1.21	Protein folding
STM0158	acnB	bifunctional aconitate hydratase	0.002	1.51	0.004	1.47	Carbohydrate utilization
STM4273	actP	acetate permease	0.031	1.50	0.010	1.65	Carbohydrate transport
STM0684	nagB	glucosamine-6-phosphate deaminase	0.021	1.50	0.121	1.45	Carbohydrate utilization

Genes downregulated by 50% or more upon fosmidomycin exposure ($p<0.05$)

			Fosmidomycin challe		Kanamycin challenge		
Name	Gene Name	Predicted Gene Product	p value	Fold change upon exposure	p value	Fold change upon exposure	Putative Role
STM1914	hcp	hydroxylamine reductase	0.001	0.03	0.001	0.02	Oxidoreductase
STM0937	ygbA	hypothetical protein	0.020	0.15	0.013	0.14	Unknown
STM4537	hcr	HCP oxidoreductase, NADH-dependent	0.043	0.19	0.045	0.20	Oxidoreductase
STM2860	hilA	invasion protein regulator	0.010	0.22	0.006	0.13	Virulence
STM0936	null	putative DNA-binding protein	0.006	0.24	0.005	0.20	DNA regulation
STM2247	potE	putrescine transporter	0.008	0.25	0.006	0.12	Polyamine transport
STM3812	speF	ornithine decarboxylase	0.001	0.25	0.015	0.19	Polyamine synthesis
STM3579	napC	cytochrome c-type protein NapC	0.000	0.25	0.007	0.18	Nitrate utilization
STM3146	invA	needle complex export protein	0.021	0.26	0.022	0.17	Flagellar biosynthesis
STM4301	nrfE	formate-dependent nitrite reductase	0.001	0.26	0.007	0.15	Nitrate utilization
STM1978	null	putative dehydrogenase	0.007	0.27	0.103	0.76	Dehydrogenases
STM2894	ycaD	putative MFS family transporter protein	0.000	0.27	0.003	0.27	Transporters
STM1834	invI	needle complex assembly protein	0.011	0.28	0.005	0.21	Flagellar biosynthesis
STM1594	invG	outer membrane secretin precursor	0.017	0.28	0.011	0.19	Cell wall components/synthesis
STM2876	invE	invasion protein	0.021	0.28	0.007	0.18	Virulence
STM3475	napH	quinol dehydrogenase membrane component	0.000	0.28	0.006	0.20	Nitrate utilization
STM3145	napB	citrate reductase cytochrome c-type subunit	0.006	0.28	0.003	0.16	Organic acid utilization
STM4315	iagB	invasion protein precursor	0.013	0.28	0.010	0.20	Virulence
STM4417	ompW	outer membrane protein W	0.002	0.29	0.017	0.19	Cell wall components/synthesis
STM0700	invF	invasion regulatory protein	0.016	0.29	0.005	0.17	Virulence
STM0701	ydfZ	putative cytoplasmic protein	0.008	0.29	0.000	0.18	Unknown
STM3846.s	cadB	lysine/cadaverine antiporter	0.010	0.30	0.042	0.29	Amino acid transport
STM1912	nrfD	putative formate-dependent nitrate reductase	0.001	0.31	0.004	0.22	Nitrate utilization
STM2869	ccmB	heme exporter protein	0.000	0.31	0.002	0.24	Transporters

STM2249	ccmB	heme exporter protein	0.000	0.31	0.003	0.24	Transporters
STM1922	null	putative outer membrane protein	0.001	0.32	0.469	0.95	Cell wall components/synthesis
STM2255	cadA	lysine decarboxylase 1	0.005	0.32	0.043	0.19	Amino acid metabolism
STM3840	pagP	palmitoyl transferase	0.004	0.32	0.113	0.60	Acyltransferases
STM2896	null	putative cytoplasmic protein	0.005	0.32	0.002	0.24	Unknown
STM1956	spaP	surface presentation of antigens protein SpaP	0.031	0.32	0.014	0.24	Cell wall components/utilization
STM4339	spaQ	needle complex export protein	0.025	0.32	0.014	0.25	Flagellar biosynthesis
STM3152	fdnI	formate dehydrogenase-N subunit gamma	0.022	0.33	0.008	0.21	Organic acid utilization
STM1798	napG	quinol dehydrogenase periplasmic component	0.003	0.33	0.005	0.21	Nitrate utilization
STM3151	invH	needle complex outer membrane lipoprotein	0.020	0.34	0.003	0.17	Flagellar biosynthesis
STM0068	nrfG	formate-dependent nitrite reductase complex	0.001	0.34	0.012	0.28	Nitrate utilization
STM4533	nrfC	putative formate-dependent nitrite reductase	0.001	0.35	0.010	0.16	Nitrate utilization
STM4281	null	putative ABC-type transport system ATPase	0.048	0.35	0.077	0.37	transporter
STM0113	null	putative anaerobic dehydrogenase component	0.003	0.35	0.016	0.25	Dehydrogenases anerobic electron transport
STM3243	invJ	needle length control protein	0.035	0.36	0.038	0.31	Flagellar biosynthesis
STM1808	null	putative sialic acid transporter	0.000	0.36	0.762	1.01	Carbohydrate transport/phosphorlyation
STM4278.S	ccmC	heme exporter protein	0.002	0.36	0.004	0.27	Transporters
STM0781	null	putative outer membrane protein	0.015	0.36	0.062	0.46	Cell wall components/synthesis
STM2873	dmsB	anaerobic dimethyl sulfoxide reductase subunit	0.010	0.37	0.024	0.31	Thiol utilization
STM1981	fdnH	formate dehydrogenase-N beta subunit	0.011	0.37	0.005	0.24	Organic acid utilization
STM0969	invB	secretion chaperone	0.034	0.37	0.008	0.23	Protein secretion
STM1133	ccmC	heme exporter protein	0.002	0.37	0.004	0.29	transporter
STM0968	spaO	surface presentation of antigens protein SpaO	0.032	0.39	0.013	0.31	Cell wall components
STM1146	dmsC	anaerobic dimethyl sulfoxide reductase subunit	0.003	0.39	0.028	0.31	Thiol utilization
STM2893	virK	virulence protein	0.006	0.39	0.427	0.84	Virulence
STM3006	null	N-acetylneuraminic acid mutarotase	0.008	0.40	0.978	0.99	Cell wall components/synthesis
STM1977	ybjM	putative inner membrane protein	0.006	0.41	0.044	0.43	Cell wall components/synthesis
STM2898	hilD	invasion protein regulatory protein	0.005	0.41	0.002	0.23	Virulence
STM2897	hypA	hydrogenase nickel incorporation protein HybF	0.001	0.42	0.005	0.34	hydrogenase
STM2257	null	putative glucosamine-fructose-6-phosphate	0.039	0.42	0.080	0.58	Carbohydrate utilization
STM2256	orgC	putative cytoplasmic protein	0.009	0.42	0.027	0.56	Unknown
STM1968	ccmF	cytochrome c-type biogenesis protein	0.009	0.43	0.002	0.21	Nitrate utilization
STM2877	null	cold shock-like protein	0.021	0.43	0.680	0.92	Protein folding
STM1732	invC	ATP synthase SpaL	0.026	0.43	0.013	0.37	Protein secretion
STM1980	orgB	needle complex export protein	0.010	0.43	0.027	0.57	Flagellar biosynthesis
STM1919	ccmF	cytochrome c-type biogenesis protein	0.007	0.43	0.003	0.22	Nitrate utilization
STM2899	null	putative cytoplasmic protein	0.032	0.44	0.215	0.62	Unknown
STM4538	nrfB	cytochrome c nitrite reductase pentaheme	0.007	0.44	0.019	0.22	Nitrate utilization
STM1509	ybgE	hypothetical protein	0.009	0.44	0.003	0.33	Unknown
STM3143	null	putative dimethylsulfoxide reductase	0.012	0.44	0.080	0.60	Thiol utilization
STM4295	prgI	needle complex major subunit	0.037	0.44	0.013	0.31	Flagellar biosynthesis
STM2558	ymdA	putative periplasmic protein	0.010	0.45	0.023	0.42	Unknown
STM4280	hybG	hydrogenase 2 accessory protein HypG	0.014	0.45	0.002	0.35	hydrogenase
STM4524	ybiP	putative integral membrane protein	0.031	0.45	0.309	0.65	Cell wall components/synthesis
STM1921	ybgT	putative outer membrane lipoprotein	0.008	0.46	0.005	0.26	Cell wall components/synthesis
STM3148	mgtC	Mg2+ transport protein	0.014	0.46	0.672	0.86	Metal ion transport

STM4269	null	putative PTS permease	0.004	0.46	0.002	0.53	Carbohydrate transport/phosphorlyation
STM3032	ycaM	putative amino-acid transporter	0.003	0.47	0.040	0.61	Amino acid transport
STM1147	null	putative PTS permease	0.024	0.47	0.008	0.50	Carbohydrate transport/phosphorlyation
STM2253	ccmH	putative heme lyase subunit	0.032	0.47	0.012	0.27	Cofactor/coenzyme biosynthesis
STM3818	ccmH	putative heme lyase subunit	0.018	0.47	0.013	0.27	Cofactor/coenzyme biosynthesis
STM1131	null	putative cytoplasmic protein	0.007	0.47	0.031	0.59	Unknown
STM3155	flhB	flagellar biosynthesis protein FlhB	0.006	0.47	0.010	0.51	Flagellar biosynthesis
STM0940	null	putative PTS permease	0.032	0.47	0.003	0.52	Carbohydrate transport/phosphorlyation
STM4536	hybE	hydrogenase 2-specific chaperone	0.011	0.47	0.003	0.40	Protein folding
STM2559	null	putative methyl-accepting chemotaxis protein	0.001	0.48	0.006	0.46	Chemotaxis
STM2799	fliR	flagellar biosynthesis protein FliR	0.001	0.48	0.011	0.51	Flagellar biosynthesis
STM1582	glnA	glutamine synthetase	0.027	0.49	0.580	0.93	Amino acid biosynthesis
STM1173	ygiH	putative glycerol-3-phosphate acyltransferase	0.016	0.49	0.479	0.80	Acyltransferases
STM0628	yhhQ	hypothetical protein	0.010	0.50	0.076	0.50	Unknown
STM0743	dcuB	anaerobic C4-dicarboxylate transporter	0.001	0.50	0.024	0.35	Carbohydrate transport/phosphorlyation
STM3244	nirD	nitrite reductase small subunit	0.004	0.51	0.081	0.44	Nitrate utilization
STM1975	flhE	flagellar protein	0.002	0.51	0.003	0.66	Flagellar biosynthesis
STM0964	ycgR	putative inner membrane protein	0.002	0.51	0.024	0.50	Cell wall components/synthesis
STM1957	caiF	DNA-binding transcriptional activator CaiF	0.023	0.51	0.015	0.33	DNA transcription
STM1475	tsr	methyl-accepting chemotaxis protein I	0.000	0.52	0.007	0.48	Chemotaxis
STM3610	fliE	flagellar hook-basal body protein FliE	0.001	0.52	0.002	0.56	Flagellar biosynthesis
STM1970	cheM	methyl accepting chemotaxis protein II	0.006	0.52	0.053	0.52	Chemotaxis
STM2949	null	putative PTS permease	0.039	0.52	0.003	0.55	Carbohydrate transport/phosphorlyation
STM0153	tdcB	threonine dehydratase	0.012	0.53	0.016	0.30	Amino Acid Degradation
STM4313	hybB	putative hydrogenase 2 b cytochrome subunit	0.000	0.53	0.017	0.27	Hydrogenase
STM3764	rmf	ribosome modulation factor	0.000	0.53	0.010	0.68	Protein synthesis
STM2890	null	putative glucosamine-fructose-6-phosphate	0.046	0.53	0.018	0.58	Carbohydrate utilization
STM1574	cydB	cytochrome d terminal oxidase polypeptide	0.003	0.53	0.013	0.31	Electron transport
STM2889	null	putative methyl-accepting chemotaxis protein	0.003	0.53	0.006	0.32	Chemotaxis
STM1568	hybD	hydrogenase 2 maturation endopeptidase	0.002	0.54	0.001	0.34	Hydrogenase
STM2258	yebN	hypothetical protein	0.018	0.54	0.180	0.59	Unknown
STM1939	blc	outer membrane lipoprotein Blc	0.006	0.54	0.001	0.60	Cell wall components/synthesis
STM2900	yghW	putative cytoplasmic protein	0.027	0.54	0.036	0.58	Unknown
STM2305	tdcC	threonine/serine transporter TdcC	0.019	0.54	0.019	0.36	Amino Acid Transporter
STM3242	fliQ	flagellar biosynthesis protein FliQ	0.021	0.54	0.043	0.68	Flagellar biosynthesis
STM1955	adiY	transcriptional activator	0.048	0.55	0.007	0.57	DNA transcription
STM4282	null	putative cytoplasmic protein	0.002	0.56	0.030	0.50	Unknown
STM2031	stpA	DNA binding protein, nucleoid-associated	0.044	0.56	0.523	1.15	DNA transcription
STM1979	fliL	flagellar basal body-associated protein FliL	0.003	0.56	0.014	0.60	Flagellar biosynthesis
STM1963	yhjG	putative inner membrane protein	0.012	0.56	0.016	0.54	Cell wall components/synthesis
STM2033	tdcD	propionate/acetate kinase	0.006	0.56	0.050	0.35	Amino acid degradation
STM1242	fliP	flagellar biosynthesis protein FliP	0.009	0.56	0.012	0.57	Flagellar biosynthesis
STM4279	envE	putative envelope protein	0.009	0.56	0.125	0.80	Cell wall components
STM1259	hypE	putative hydrogenase formation protein	0.010	0.57	0.113	0.49	Oxidoreductases
STM2294	trg	methyl-accepting chemotaxis protein III	0.013	0.58	0.013	0.51	Chemotaxis
STM2865	flhD	transcriptional activator FlhD	0.018	0.58	0.020	0.41	DNA transcription

STM3479	mltD	membrane-bound lytic murein transglycosylase D	0.039	0.58	0.217	0.81	Cell wall components/synthesis
STM2858	flhA	flagellar biosynthesis protein FlhA	0.021	0.58	0.039	0.72	Flagellar biosynthesis
STM2213	cbiA	cobyrinic acid a,c-diamide synthase	0.021	0.58	0.023	0.36	Cofactor biosynthesis
STM4308	yfiN	hypothetical protein	0.019	0.58	0.021	0.62	Unknown
STM0081	tdcA	DNA-binding transcriptional activator TdcA	0.016	0.58	0.067	0.36	Amino acid degradation
STM1183	iap	alkaline phosphatase isozyme conversion	0.019	0.58	0.039	0.69	Kinase/phosphorylase
STM2892	hilC	invasion regulatory protein	0.012	0.58	0.001	0.37	Virulence
STM3072	cbiT	cobalt-precorrin-6Y C(15)-methyltransferase	0.016	0.59	0.019	0.52	Cofactor biosynthesis
STM4535	infA	translation initiation factor IF-1	0.016	0.59	0.068	0.70	Protein synthesis
STM0740	folB	bifunctional dihydroneopterin	0.009	0.59	0.092	0.80	Cofactor biosynthesis
STM2528	smvA	methyl viologen resistance	0.024	0.59	0.216	0.76	Transport
STM1066	null	putative ABC-type cobalt transport system	0.008	0.59	0.419	0.71	Metal ion transport
STM1243	tnpA_2	transposase for IS200	0.032	0.59	0.098	0.64	DNA modification
STM1626	fliZ	flagella biosynthesis protein FliZ	0.001	0.59	0.005	0.47	Flagellar biosynthesis
STM4340	cbiE	cobalt-precorrin-6Y C(5)-methyltransferase	0.018	0.60	0.044	0.51	Cofactor biosynthesis
STM1132	avrA	secreted effector protein	0.030	0.60	0.001	0.31	Protein secretion
STM3817	null	putative inner membrane protein	0.038	0.60	0.095	0.72	Cell wall components
STM1925	flgD	flagellar basal body rod modification protein	0.003	0.60	0.031	0.73	Flagellar biosynthesis
STM1328	ygdK	putative FeS center assembly protein	0.014	0.61	0.373	0.80	Cofactor/coenzyme biosynthesis
STM0260	fliH	flagellar assembly protein H	0.021	0.61	0.049	0.71	Flagellar biosynthesis
STM1176	hsdM	DNA methylase M	0.015	0.61	0.340	0.66	DNA synthesis/modification
STM1913	fliO	flagellar biosynthesis protein FliO	0.049	0.61	0.076	0.65	Flagellar biosynthesis
STM2314	leuA	2-isopropylmalate synthase	0.049	0.61	0.581	0.84	Amino Acid Biosynthesis
STM0965	yeiU	putative permease	0.011	0.61	0.286	0.82	Transport
STM1569	null	putative inner membrane protein	0.012	0.61	0.034	0.79	Cell wall components
STM3075	cydA	cytochrome d terminal oxidase polypeptide	0.008	0.61	0.028	0.42	Electron transport
STM2024	frdD	fumarate reductase subunit D	0.017	0.61	0.041	0.59	Organic acid utilization
STM2035	flgG	flagellar basal body rod protein FlgG	0.000	0.61	0.028	0.66	Flagellar biosynthesis
STM2895	ynaI	putative integral membrane protein	0.007	0.62	0.111	0.70	Cell wall components
STM2672	fliK	flagellar hook-length control protein	0.034	0.62	0.074	0.71	Flagellar biosynthesis
STM1941	rstA	DNA-binding transcriptional regulator RstA	0.035	0.62	0.330	0.86	DNA transcription
STM3216	fliG	flagellar motor switch protein G	0.000	0.62	0.001	0.69	Flagellar biosynthesis and motility
STM1573.Sc	yfaZ	putative inner membrane protein	0.013	0.62	0.146	0.76	Cell wall components/synthesis
STM2252	yobG	hypothetical protein	0.001	0.62	0.058	0.79	Unknown
STM3207	nrfA	cytochrome c552	0.000	0.62	0.079	0.32	Nitrite reduction
STM3245	fliA	flagellar biosynthesis sigma factor	0.001	0.62	0.002	0.51	Flagellar biosynthesis
STM2891	srfB	putative virulence protein	0.036	0.62	0.107	0.73	Virulence
STM0966	null	putative transcriptional regulator	0.017	0.62	0.382	0.92	DNA regulation
STM2936	motB	flagellar motor protein MotB	0.001	0.63	0.011	0.50	Flagellar biosynthesis
STM4539	modA	molybdate transporter periplasmic protein	0.004	0.63	0.024	0.52	Metal ion transport
STM2781	ygdQ	putative transport protein	0.012	0.63	0.015	0.61	Transport
STM2867	fliN	flagellar motor switch protein FliN	0.006	0.63	0.002	0.59	Flagellar biosynthesis
STM4525	hsdS	type I restriction enzyme specificity protein	0.014	0.63	0.041	0.69	DNA binding
STM1840	cheA	chemotaxis protein CheA	0.006	0.64	0.048	0.59	Chemotaxis
STM0834	dmsA	anaerobic dimethyl sulfoxide reductase subunit	0.005	0.64	0.046	0.43	Thiol utilization
STM1911	null	putative glucose-6-phosphate dehydrogenase	0.035	0.64	0.019	0.77	Pentose phosphate pathway

STM0946	amyA	cytoplasmic alpha-amylase	0.025	0.64	0.008	0.58	Carbohydrate ultilization
STM2985	tnpA_5	transposase for IS200	0.023	0.64	0.079	0.64	DNA modification
STM1130	null	putative cytoplasmic protein	0.019	0.65	0.008	0.62	Unknown
STM0871	pipB2	secreted effector protein	0.036	0.65	0.136	0.80	Protein secretion
STM4007	tnpA_1	transposase	0.035	0.65	0.092	0.64	Nucleic acid binding
STM0741	yjcE	Na/H transport protein	0.033	0.65	0.405	0.86	Metal ion transport
STM2875	null	hypothetical protein	0.040	0.65	0.071	0.62	Unknown
STM3144	ybjX	VirK-like protein	0.024	0.65	0.179	0.75	Virulence
STM1179	ptpS	putative 6-pyruvoyl tetrahydrobiopterin	0.031	0.65	0.717	0.94	Cofactor biosynthesis
STM1663	cbiL	cobalt-precorrin-2 C(20)-methyltransferase	0.015	0.66	0.097	0.69	Cofactor biosynthesis
STM2838.S	menE	O-succinylbenzoic acid--CoA ligase	0.029	0.66	0.119	0.74	Acyltransferases
STM4540.S	null	putative reverse transcriptase	0.032	0.66	0.131	0.80	Unknown
STM1971	tnpA_4	transposase	0.033	0.66	0.138	0.71	DNA modification
STM1923	aceF	dihydrolipoamide acetyltransferase	0.034	0.66	0.002	0.48	Acyltransferases
STM1974	cbiC	cobalt-precorrin-8X methylmutase	0.028	0.66	0.027	0.47	Cofactor biosynthesis
STM2868	null	putative secreted protein	0.030	0.66	0.650	0.92	Protein secretion
STM2030	flgK	flagellar hook-associated protein FlgK	0.012	0.66	0.043	0.55	Flagellar biosynthesis
STM1300	nhoA	putative arylamine N-acetyltransferase	0.036	0.67	0.031	0.72	Acyltransferases
STM4277	null	putative chemotaxis signal transduction protein	0.019	0.67	0.021	0.42	Chemotaxis
STM0742	rnpA	ribonuclease P	0.024	0.67	0.437	0.83	RNA degradation
STM0953	flgA	flagellar basal body P-ring biosynthesis protein	0.015	0.67	0.060	0.77	Flagellar biosynthesis
STM2780	gutQ	D-arabinose 5-phosphate isomerase	0.005	0.67	0.981	1.00	Carbohydrate utilization
STM3206	motA	flagellar motor protein MotA	0.010	0.67	0.005	0.47	Flagellar biosynthesis
STM3814	null	putative periplasmic protein	0.007	0.67	0.016	0.52	Unknown
